# Industrial Production of Bioactive Nutrient-Enhanced Extra Virgin Olive Oil under Continuous-Flow Ultrasound and Pulsed Electric Field Treatment

**DOI:** 10.3390/foods13162613

**Published:** 2024-08-20

**Authors:** Luisa Boffa, Emanuela Calcio Gaudino, Giorgio Grillo, Arianna Binello, Giorgio Capaldi, Duarte Rego, Marcos Pereira, Giancarlo Cravotto

**Affiliations:** 1 Department of Drug Science and Technology, University of Turin, Via P. Giuria 9, 10125 Turin, Italy; luisa.boffa@unito.it (L.B.); emanuela.calcio@unito.it (E.C.G.); giorgio.grillo@unito.it (G.G.); arianna.binello@unito.it (A.B.); giorgio.capaldi@unito.it (G.C.); 2 EnergyPulse Systems, Est Paco Lumiar Polo Tecnológico Lt3, 1600-546 Lisbon, Portugal; duarte.rego@energypulsesystems.com (D.R.); marcos.pereira@energypulsesystems.com (M.P.)

**Keywords:** flow process, ultrasound, pulsed electric field, extra virgin olive oil (EVOO), polyphenols, tocopherols, green Coratina, industrial scale, accelerated shelf-life

## Abstract

Extra virgin olive oil (EVOO) is a cornerstone of the Mediterranean diet. Many studies have highlighted its crucial preventive role against cardiovascular disease, neurodegenerative disorders, metabolic syndrome and cancer, with these effects being due to the synergistic anti-inflammatory and antioxidant activities of minor components, such as polyphenols and tocols. The aim of the present study is to implement new technologies for olive oil mills and develop an efficient large-sized industrial process for the continuous extraction of healthier EVOOs that are enriched with these bioactive compounds. Non-thermal technologies, namely ultrasound (US) and pulsed electric field (PEF), have been tested, separately and in combination, to eliminate the need for traditional malaxation. There is extensive literature to support the efficacy of ultrasound-assisted extraction (UAE) and PEF treatments in EVOO production. A newly designed US device and a PEF industrial chamber have been combined into a single, integrated continuous-flow setup, the performance of which in the extraction of EVOO from green Coratina olives has been evaluated herein. Extraction yields, physico-chemical and organoleptic characteristics, and polyphenol and tocol contents were monitored throughout the trials, and the last three were measured at accelerated aging times (AAT) of 15 and 30 days. The US and combined US-PEF processes not only increased daily oil production (ton/day, by nearly 45%), but also eliminated the need for kneading during malaxation, resulting in significant energy savings (approximately 35%). In addition, these innovations enriched the resulting EVOO with nutritionally relevant minor components (8–12% polyphenols, 3–5% tocols), thereby elevating its quality and market value, as well as overall stability. The introduction of continuous-flow US and PEF technologies is a remarkable innovation for the EVOO industry, as they offer benefits to both producers and consumers. The EVOO resulting from non-thermal continuous-flow production meets the growing demand for healthier, nutrient-enriched products.

## 1. Introduction

Due to its positive effects on human health, the importance of consuming extra virgin olive oil (EVOO) as part of the human diet is now universally recognized, with the associated benefits mainly being the reduced risk of cardiovascular disease, neurodegenerative disorders and cancer [1,2]. EVOO consists mainly of fatty acids (~98%), of which up to 85% are unsaturated (e.g., oleic, linoleic, and palmitoleic acids) and about 14% are saturated (e.g., palmitic and stearic acids), in the form of glycerides (defined as the saponifiable fraction), while the minor components (the unsaponified fraction) must not exceed 2% of the total [3,4]. Their anti-amyloid and antioxidant activities mean that secondary metabolites, such as phenols and tocopherols, are thought to play a crucial role in both the nutritional properties and biological activity of this valuable food [5]. This is also highlighted in one of the health claims for olive oil, as approved by the European Food Safety Authority (EFSA): “olive oil polyphenols contribute to the protection of blood lipids from oxidative stress”, which refers to a sample containing at least 5 mg of hydroxytyrosol and its derivatives (such as the oleuropein complex and tyrosol) per 20 g of olive oil [6,7,8]. Moreover, European Regulation n. 432/2012 classifies olive oils according to their impact on health, specifying that phenolic compound content must reach at least 250 mg/kg in order to protect blood lipids from oxidative stress [9].

In particular, this heterogeneous class of compounds consists of hydroxytyrosol and tyrosol (simple phenols), oleuropein and ligstroside aglycones and their aldehydic forms (secoiridoids), the dialdehydic forms of hydroxytyrosol-linked decarboxymethyl elenolic acid (e.g., oleacein) and tyrosol-linked decarboxymethyl elenolic acid (e.g., oleocanthal), 1-acetoxypinoresinol and pinoresinol (lignans), acetylated hydroxytyrosol, luteolin and apigenin (flavonoids), p-coumaric acid and vanillic acid (phenolic acids) [10]. The polyphenol content in olive oil depends on its variety, the month of harvest and the ripeness of the fruit as well as the extraction and storage processes and has been reported to lie between 50 and 1000 mg/kg, with the highest values observed in EVOO [11,12].

As defined by the International Olive Council (IOC), it has unique physico-chemical and organoleptic properties and is derived from the mechanical extraction of the fruit of the *Olea europaea* L. tree, without any treatment other than washing, decanting, centrifuging and filtering [13]. EVOO is mostly associated with the Mediterranean area and about 70 per cent of olive production takes place in the European Union. It is therefore an agro-industrial product of high socio-economic impact and one that requires the constant attention of regulatory and control agencies if its quality and safety for the consumer are to be ensured [14]. Commonly, olive oil is produced by small companies with production lines that can provide 0.4 to 2 ton/h of olive paste, although there are also larger plants that allow a throughput of 12 ton/h thanks to multiple lines [15]. More specifically, after the collection, selection and washing of the olives, the crushing phase produces a paste, which is then subjected to the malaxing process. The oil is then extracted using hydraulic presses and separated from the aqueous phase via centrifugation and subsequently filtered [16].

Malaxation is considered one of the most important steps in the production of olive oil, as it determines the balance between the quality and quantity of the product obtained. Increasing the temperature during this process (up to 30 °C) improves the extraction yield by stimulating enzymatic activity, reducing the viscosity of the olive paste and thus favouring adequate coalescence of the small oil droplets [17]. The malaxation, which usually lasts 45–60 min, can also be extended to maximise the quantity of oil. However, the organoleptic and nutritional properties (e.g., antioxidant activity) of the oil can be affected by variations in the malaxing parameters. Numerous studies have been carried out to investigate these parameters, which represent reference conditions for EVOO production [18]. While the crushing and separation stages are continuous processes, malaxation is a batch-size process and often becomes a bottleneck, slowing down the entire oil production [19]. Furthermore, due to a poor ratio between its large volume and tiny surface area, the malaxer unit has a low heat transfer coefficient and is an inefficient heat exchanger. Unfortunately, oil mills must either reduce oil production or purchase more malaxation equipment. Both options affect the overall cost of the process [20,21].

As indicated in European research programs, it is important to investigate the application of emerging technologies in the design of innovative and sustainable processes aimed at increasing yields in the production of EVOO, preserving (or improving) its nutritional values and organoleptic characteristics, while reducing processing time and energy consumption. Although this may seem to diverge from the definition of EVOO as a traditional food product, there is now growing consumer interest in products whose health properties have been improved without altering their traditional features [22].

Pulsed electric field (PEF), heat exchangers, high pressure processing and, in particular, ultrasound (US), among others, are promising technologies that can improve EVOO production [23,24,25,26,27]. A number of studies have evaluated the scalability of pilot plants of such technologies to an industrial scale, involving medium-sized olive oil producers [15].

In order to obtain EVOO with enhanced antioxidant and anti-inflammatory properties, it is important to pursue processing operations that allow the enrichment of the phenolic compound content, and then improve their extraction from the olive paste. In general, innovative processes focus on malaxation, as this is the fundamental phase in which the main transformations take place, and is therefore responsible not only for the final composition/taste of the oil, but also for the possible degradation of biologically active substances. In this context, there are still few literature data concerning both the shelf-life and evaluation of trained sensory panels for oils produced with innovative technologies. It is also important to assess the cost of the extraction plants, as, although the increase in EVOO quality and the sustainability of the process may be appreciated, a concomitant increase in the price of the final product is generally not welcomed by consumers [18]. In addition, the return-on-investment analysis is an important parameter for the manufacturer to justify the possible acquisition of equipment.

The cavitation phenomenon of US [28] has been reported to improve the extraction yields of EVOO without significantly affecting its legal parameters [13,29]. Due to the enhanced mass transfer, cavitation can mimic the physical effects of malaxation on the olive paste. Additionally, US can enhance the bioactive migration from the aqueous phase to the oily one, together with the coalescence of the latter. However, as the heating exerted may have a negative impact on minor constituents, processing parameters must be carefully modulated [28,30]. Thanks to the combination of the US effect and the modulation of the heat exchange of the olive paste, it is possible to design an efficient, continuous process on an industrial scale, eliminating the need for the traditional batch malaxation phase [31,32].

Similar considerations can be made for PEF, as the effect of electroporation means that the extraction of oil and minor compounds (e.g., phenolic and volatile components) from olives is favoured by the efficient rupture of cell membranes [33]. As a result, the non-thermal PEF technology can provide improved oil yields and reduced process times using less intense conventional extraction parameters, such as temperature and malaxation time [34]. The potential of using PEF has been evaluated both after crushing, to increase oil release, and after malaxation to promote de-emulsification and mass transfer during the centrifugation phase [15]. On an industrial scale, a continuous system using PEF as pre-treatment prior to oil separation has reported improved extraction yields and functional component content, without altering the chemical-physical and sensory parameters (in terms of defects and off-flavours) of EVOO [35].

Olive oils (OOs) must comply with different rules and standards specified by several organizations, such as the European Union (EU), the IOC and the Codex Alimentarius (2017) [36].

Regulation (EEC) 2568/91 was the cornerstone of all EU legislation on OO, establishing the mandatory official parameters to be checked for OO quality and purity, the limits for each parameter and commercial category of OO [29]. Moreover, it described official analytical methods required for the assessment of the commercial category of OO. In 2022, it was repealed by Commission Delegated Regulation (EU) 2022/2104 with the last revision in 2024 (Commission Delegated Regulation (EU) 2024/1401) [37,38]. Outside the EU, IOC standards apply, with a reference document for any member country, last revised in 2022 [13]. Since the EU is a member of the IOC, it harmonized its regulation to IOC Trade Standard. Considered a worldwide scenario, edible OOs, as most foods, conform to the standard developed by Codex Alimentarius (CODEX STAN 33–1981, reviewed in 2017, last amended in 2021) with some essential purity and quality characteristics fixed as mandatory [39].

The identity characteristics include purity and quality criteria, applicable to olive oils and olive pomace oils. Purity and authenticity parameters are represented by fatty acids (FAs), sterol and triterpene dialcohol composition, *trans* FAs, wax, maximum difference between the actual and theoretical ECN 42 triacylglycerol (TAG), stigmastadiene and 2-glyceryl monopalmitate content, and unsaponifiable matter. Quality parameters include organoleptic characteristics, free acidity, peroxide value (PV), UV-specific extinctions (K232, K268/270, ΔK), FA ethyl ester content, moisture and volatiles, insoluble impurities, flash point, trace metals, and, finally, tocopherol and polyphenol content. The quality criteria mainly concern the quality of the olives, the state of preservation of the olive fats (fermentation, oxidation processes) and their modification by the technological processes used in the production of OO, while the purity criteria prove the authenticity of the olive oils, possible refining or fraudulent physico-chemical processes or adulteration. All these parameters are not only relevant to the classification of OOs, but can also be used to assess their shelf life.

The assessment of the stability of an OO, especially of EVOO, is a current issue for food manufacturers, not only in terms of the need to comply with EU regulations throughout the product lifecycle, but also to preserve consumer confidence. EVOO is constantly exposed to degradation processes, especially hydrolytic and oxidative reactions, which affect its FA, tocopherol and phenolic composition. External factors such as light, temperature, and oxygen, as well as other pro-oxidants (such as chlorophylls) affect EVOO by increasing oxidation kinetics. Auto-oxidation phenomena have been reported to occur naturally in EVOO even under controlled conditions [40].

There are currently four authorised health claims that can be used on EVOO labels for commercial reasons, in accordance with EC Regulation 1924/2006 (art. 13) [41]. EU Regulation n. 432/2012 sets out a list of permitted health claims for food with the exception of claims “referring to the reduction of disease risk and to the development and health of children” [9]. As cited above, the use of these health claims requires a concentration of phenolics (hydroxytyrosol, tyrosol and complex derivatives) in the EVOO of more than 250 mg/kg (5 mg/20 g), more than 3 g/kg (0.3 g/100 g) of α-linolenic acid and more than 18 mg/kg (1.8 mg/100 g) of vitamin E (α-tocopherol) throughout the shelf life of the EVOO [10].

Free fatty acids (FFA), PV, UV-specific extinctions (K232, K270, ΔK), 1,2-diacylglycerols, pyropheophytins, sensory analysis, induction time, total phenolics, α-tocopherol and volatiles are the most commonly used chemical quality parameters for olive oil shelf life in the literature. All these parameters, except the last four, have established limits within the quality standards of the IOC [42,43,44,45]. EVOO, especially those with high initial phenolic content, have shown high stability during storage with regards to EU extra virgin quality parameters (PV, K232, K270) [46,47,48,49]. However, few studies have been performed on the degradation of polar phenolics, FAs and tocopherols over the course of EVOO shelf life to investigate whether these health claims continue to be met. Accelerated Shelf Life Testing (ASLT) is often suggested as a means of accelerating shelf-life evaluation [50,51]. When properly applied, the ASLT method allows the estimation of the shelf life of a product under storage conditions commonly experienced in the marketplace by modelling data obtained under accelerated storage conditions. Temperature is certainly the most common accelerating factor used in ASLT, and the shelf life at the temperature of interest is predicted using the Arrhenius equation, which can ultimately be considered a shelf-life prediction model [50,52].

The ASLT approach has been effectively applied by Mancebo-Campos et al. [10], to study the quality evolution of EVOO stored in open amber glass containers at increasing temperatures from 25 to 60 °C in order to evaluate the oxidative effect of temperature on minor components (phenols, tocopherol, pigments) and to properly complete a model for predicting storage life. The linear Arrhenius model was found to suitably describe the temperature-dependent kinetics of phenolic compounds and α-tocopherol [26,46].

Our previous trials have aimed to assess the efficiency of US and PEF, used both alone and in synergistic combination (US-PEF), in a pilot scale and medium-sized olive oil mill working in continuous flow (500–900 kg/h process flow rate, olives of Coratina cultivar at half ripening stage). The hybrid US-PEF system provided the best results in terms of extractive yield and the quality of the virgin olive oil without traditional batch malaxation and the addition of water into the decanter [53]. Based on these preliminary results, this work focuses on the transfer of the US and PEF technologies to large-sized industrial scale (1.5–2 ton/h process flow rate, up to 50 ton/day) extraction of EVOO from green Coratina olives, increasing micronutrient content and leaving its quality characteristics unaffected. The main goal is to remove the bottleneck of the traditional batch malaxation, the only step that cannot be performed in flow mode, thus reducing the overall productivity of the plant and potentially affecting the oil quality. To this end, US with its mechanical action has been exploited as the first agent, followed by PEF, which, by reducing the oil dispersion prior to the decanting step, can enhance the recovery efficiency.

In its second part, this work evaluates the nutritional profile of the EVOO obtained, paying attention to the health claims, and the evolution of the associated nutrients over time using an accelerated aging method (storage in amber glass in the light at 40 °C for 15 and 30 days).

Finally, the industrial scalability of the proposed extractive protocol has been evaluated in terms of economic feasibility.

## 2. Materials and Methods

### 2.1. Olive Cultivar

The green Coratina olives (3 November 2022) were purchased from Frantoio Gandolfo (Imperia, Italy).

### 2.2. Sustainable Industrial Production of EVOO: Description of the Devices Used

#### 2.2.1. Conventional Equipment

**Figure 1:**
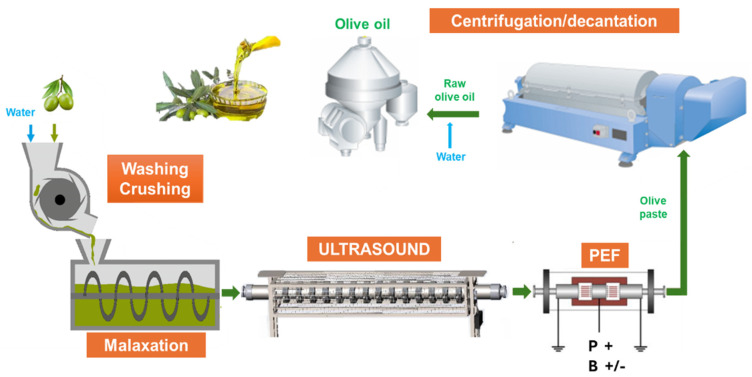
Flow diagram of operations: 1, CTRL (malaxation step, US and PEF off); 2, US (no malaxation, PEF device off); 3, US + PEF_P trial (no malaxation, US on, PEF in positive mode); 4, US + PEF_B (no malaxation, US on, PEF in bipolar mode); 5, M_PEF+P (malaxation step, US off and PEF in positive mode).

Conventional facilities for the control trial (CTRL, **1**, Figure 1), such as the hammer crusher and downstream systems (malaxer, 3-phase decanter and vertical centrifuge), are detailed in our previous article in the Supplementary Materials [53].

#### 2.2.2. US Device

The US equipment contains an ultrasonic flow reactor (internal volume of 15.5 L, reactor chamber volume 13.5 L), working at 25 kHz (mean power 2.3–2.4 kW, with a flow rate of 1.3–1.5 t/h) (Figure S1a,b), and is equipped with a CAA-GP-1 ultrasonic generator, a laptop with specific software installed on PC and an interface converter MOXA Uport 1150. In particular, the US reactor was used for the US trial (2, with PEF device off, Figure 1), for the US + PEF_P trial (3, PEF in positive mode, Figure 1), for US + PEF_B (4, PEF in bipolar mode, Figure 1) and was switched off for the M_PEF+P trial (5, malaxation step and PEF in positive mode, Figure 1).

#### 2.2.3. PEF Equipment

The adopted PEF equipment was an EPULSUS-BM3-15 (EnergyPulse Systems, Lisbon Portugal), with maximum voltage of 15 kV and maximum power of 9 kW. The treatment chamber was a DN50 with a 50 mm gap between the electrodes (volume of 196 mL, Figure S1c). Table 1 reports the experimental setup according to the trial.

### 2.3. General Procedure for Industrial-Scale EVOO Production

The general flow diagram of the process, comprising all the stages, is presented in Figure 1. Different technologies are involved (or not considered) in the different trials as described in the following paragraphs. The yields of the EVOO produced during the various trials were first compared with those of the conventional extractive protocol (CTRL, 1 in Figure 1).

#### 2.3.1. Control Process (CTRL)

The CTRL industrial-scale process trial was performed as follows. A quantity of 1800 kg of green Coratina olives was crushed at 2500 kg/h. The adopted olive paste flow rate was 1300 kg/h. A malaxation step (30 min at 30 °C) was used and water (250 L/h) was added to the olive paste during the separation phase in the 3-phase decanter (Figure 1).

#### 2.3.2. US-Assisted Process

The US-assisted industrial-scale process trials were performed as follows. A quantity of 1800 kg of green Coratina olives was crushed at 2500 kg/h. Sonication treatment was performed on the olive paste, using a US device operating at 2.3–2.4 kW (average power) and 25 kHz (Figure 1 and Figure S1), instead of the conventional malaxation step. The olive paste was sonicated through the US device at a flow rate of 1300 kg/h and directly pumped through the PEF chamber (OFF) into the decanter. The olive paste residence time in the US reactor was 37.4 s (with the approximation Kg = L of paste), and only for that time did the olive paste reach 31 °C. Water (250 L/h) was added to the olive paste during the separation phase in the decanter. The process overview, with respective operations, is shown in Figure S1.

#### 2.3.3. US-PEF-Assisted Process

The hybrid US-PEF-assisted industrial-scale process trials were performed as reported in Section 2.3.2, with the simple addition of a PEF modulus (Figure S1c) after the US device (see Section 2.2.3), working with the parameters reported in Table 1. A quantity of 1800 kg of green Coratina olives was crushed at 2500 kg/h. The sonication treatment was performed on the olive paste, using the US device (2.3–2.4 kW and 25 kHz) (Figure 1 and Figure S1), instead of the conventional malaxation step. The olive paste was sonicated through the US device at a flow rate of 1300 kg/h and directly pumped through the PEF chamber (ON) into the decanter. The electrical conductivity of the olive paste recorded using the PEF device was 4 mS/cm. PEF was used in positive mode (US + PEF_P) for trial 3 and in bipolar mode (US + PEF_B) for trial 4. The olive paste residence time in the US and PEF units was 37.4 and 0.54 s, respectively (with the approximation Kg = L of paste), and only for that time did the olive paste reach 31 °C. Water (250 L/h) was added to the olive paste during the separation phase in the decanter. The process flow diagram, with respective operations, is shown in Figure 1.

#### 2.3.4. PEF-Assisted Process

The PEF-assisted industrial-scale process trial was performed as follows. A quantity of 1800 kg of green Coratina olives was crushed at 2500 kg/h, the adopted olive paste flow rate was 1300 kg/h. A traditional batch malaxation was performed before the PEF treatment (30 min at 30 °C). The olive paste was pumped through the US device (OFF) at a flow rate of 1300 kg/h and then through the PEF chamber (ON) into the decanter. The electrical conductivity of the olive paste recorded using the PEF device was 4 mS/cm. PEF was used in positive mode (M + PEF_P), working with the parameters reported in Table 1. The olive-paste residence time in the PEF chamber was 0.54 s (with the approximation Kg = L of paste). Water (250 L/h) was added to the olive paste during the separation phase in the 3-phase decanter. The process flow diagram, with respective operations, is shown in Figure 1.

### 2.4. General Analytical Procedures for EVOO Analysis

After production, the olive oils were evaluated in terms of quality criteria (FFA, PV, insoluble impurities, moisture and volatiles, UV-specific extinctions and organoleptic characteristics) and purity criteria (FA composition, *trans* FA isomers, sterol and triterpenic dialcohol composition). Finally, the amounts of important micronutrients, such as biophenols, tocopherols and tocotrienols, were assessed.

#### 2.4.1. Quality Parameters of Coratina Oil Samples: FFA, PV, Insoluble Impurities, Moisture and Volatiles and Organoleptic Assessment

FFA, PV, insoluble impurities, moisture and volatile matter, as well as the organoleptic assessment were determined for all the oil samples in an external certified analytical laboratory, meeting the requirements of the standard ISO/IEC 17025:2017 [54].

The official analytical methods were used for the following analyses: organoleptic assessment (COI/T.20/Doc n°15/rev.10 2018; Reg. CEE 2568/1991 with the amendments of EU Commission Implementing Regulation 1348/2013, 1227/2016 and 1604/2019) [28,55], free acidity (COI/T.20/Doc. n°34/Rev.1 2017) [56], peroxide index (COI/T.20/Doc. n°35/Rev.1 2017) [57], insoluble impurities (ISO 663:2017) [58], moisture and volatile matter (ISO 662:2016) [59].

#### 2.4.2. Quality Parameters of Coratina Oil Samples: UV-Specific Extinctions, Total Polyphenols, Tocotrienols and Tocopherols

UV spectrophotometric analyses were performed on a Cary 60 UV-Vis spectrophotometer (Agilent Technologies, Santa Clara, CA, USA), using 1 cm quartz cuvettes and iso-octane (for spectroscopy Uvasol^®^, Supelco, Merck), according to the following method: COI/T.20/Doc. No 19/Rev. 5 2019 [60].

Polyphenol HPLC analyses were performed on a Waters binary pump 1525 linked to a 2998 PDA (Waters Corp., Milford, CT, USA), using a Luna RP C18 column (250 mm, 4.6 mm, 5 µm; Phenomenex, Torrance, CA, USA). HPLC-grade solvents and analytical standards were purchased from Merck (Rome, Italy) and PhytoLab GmbH & Co. KG (Vestenbergsgreuth, Germany). The analyses were performed according to the method described in COI/T.20/Doc. No 29/Rev.1/2017 [61]. The mobile phases used 0.2% H_3_PO_4_ (A) and 1:1 MeCN/MeOH (B), the monitored wavelength was 280 nm, while three-dimensional data were acquired in the 200–400 nm range (gradient: 0 min, 4% B; 40 min, 50% B; 45 min, 60% B; 60 min, 100% B; 70 min, 100% B).

Tocopherol and tocotrienol HPLC analyses were performed on a Waters binary pump 1525 linked to a 2998 PDA (Waters), using a Spherisorb 5µ Silica column (250 mm, 4 mm, 5 µm; Waters). (±)-α-Tocopherol, rac-β-tocopherol, (+)-*γ*-Tocopherol and (+)-*δ*-tocopherol were purchased from Merck. 2-Propanol (hypergrade LC-MS LiChrosolv^®^) was purchased from Merck, while n-Hexane (HPLC Grade, 95% min) was obtained from Alfa Aesar (Haverhill, MA, USA). The analyses were performed according to the official IUPAC method [62]. A 0.5:99.5 propan-2-ol/hexane mixture was used as the mobile phase and the monitored wavelength was 292 nm.

#### 2.4.3. Purity Parameters of Coratina Oil Samples

FA composition, FA *trans* isomers, sterol composition and content and alcoholic compounds were determined for the CTRL oil sample in an external certified analytical laboratory, meeting the requirements of the standard ISO/IEC 17025:2017 [54].

Any adulteration with other oils or any other physico-chemical fraudulent processes could be excluded, as olives came from the same batch in the same day and the same configuration of the equipment was used to produce all the oil samples, modulating US and PEF units on and off. Moreover, from previous extensive data [53], it was assessed that US and PEF did not affect these purity parameters of the produced olive oils.

The official analytical methods were used for the following analyses: FA composition and *trans* isomers (COI/T.20/Doc. N°33/Rev.1 2017) [63], sterol and alcoholic compounds (COI/T.20/Doc. n°26/Rev.5 2020) [64].

#### 2.4.4. Statistical Analyses

Statistical analyses were performed using R software (R Foundation for Statistical Computing, Vienna, Austria), version 4.4.0 (24 April 2024). The analyses were all performed in triplicate. Residuals were checked for normality using the Shapiro–Wilk and Kolmogorov–Smirnov tests, and variances were checked for homogeneity using Levene’s test. Tocopherols, tocotrienols and polyphenol determination results were processed using ANOVA statistical techniques. In detail, a single-way ANOVA was performed on Coratina oil samples obtained using different techniques (Section 3.4). Tukey’s HSD post-hoc multiple comparison test was used with a confidence level of 0.95 and a significance level α = 0.05, to determine the differences between the different sample means (*p* < 0.05).

For the evaluation of EVOO shelf-life, two-way ANOVA followed by Tukey’s multiple comparisons test were performed using GraphPad Prism version 10.2.3 (403) for Windows 64-bit (GraphPad Software, Boston, MA, USA, www.graphpad.com, accessed on 21 April 24). The effects of two factors (treatment with 5 levels × time with 3 levels), and their interactions were evaluated on the total polyphenol and total tocol content of EVOOs (*p* < 0.05). Comparisons of means were run between treatments at the same time and between different times within the same treatment with a confidence level of 0.95, and a significance level α = 0.05. Residuals were checked for normality using the Shapiro–Wilk and Kolmogorov–Smirnov tests, and variances were checked using Spearman’s test for heteroscedasticity. The same test was performed with R software to give the means and groups for total polyphenol and tocol content in oil samples (Section 3.5).

#### 2.4.5. Shelf-Life Evaluations on Green Coratina Oil Samples

The shelf-life studies for the EVOO produced from the green Coratina variety (CTRL, US, US + PEF_P, US + PEF_B, M + PEF_P) were carried out at the Acesur company (Jaen, Spain) using accelerated climatic chamber conditions (40 °C with 12 h of light per day). EVOO were subjected to a filtration step (T0) before being stored in the climatic chamber for periods of 15 and 30 days (T15 and T30). At 15 days, oil samples were analysed in an external certified laboratory (organoleptic evaluation, free FA and PV), while at 30 days the organoleptic assessment was provided. Total polyphenol and tocol contents were determined by our research laboratory for both the T15 and T30 samples.

## 3. Results and Discussion

### 3.1. Technology-Assisted EVOO Production

In our previous work [53], a numbering-up strategy for US devices was applied when moving from a pilot scale to medium-sized olive oil plants. However, in order to meet the requirements of an even larger scale of industrial production, the US reactor has been redesigned and its capacity increased to boost oil productivity and, possibly, the micronutrient content of the recovered oil. Specifically, a tubular US device (about 13.5 L capacity) has been newly developed, and is tested herein for the first time, alone (US, 2 in Figure 1) and in combination with PEF, working in positive and bipolar modes (US + PEF_P and US + PEF_B, 3 and 4 in Figure 1). This setup operated at a flow rate of 1.3 ton/h of olive paste in continuous mode during the trials, but the device could easily reach 2080 kg/h with a proper decanter unit (50 ton/day). An alternative process that is based on PEF in positive mode has also been considered, but in a different set-up which sees it combined with conventional malaxation (M + PEF_P) (5, Figure 1).

Generally speaking, the use of non-conventional techniques did not significantly influence oil extraction yields from green ripening Coratina olives. The control extraction (CTRL) in an oil mill gave 14.4% of EVOO, which was equivalent to trial 5 in which a positive PEF step was inserted after malaxation (M + PEF_P). US alone (2) provided a 14% oil yield, which is similar to that given by its combination with positive PEF treatment (14.2%, 3, US + PEF_P). A slightly lower, but not statistically significant, oil yield was recovered when US was coupled with bipolar PEF (13.8%, 4, US + PEF_B).

### 3.2. Quality Assessment of Oil Samples

As shown in Table 2, all collected oil samples were classified as EVOO in each of the quality parameters evaluated, showing comparable values of free acidity, PV, insoluble impurities, moisture and volatile matter, as well as absorbency in UV (K232, 268 and ΔK), meaning that the various US and/or PEF treatments did not change the physical-chemical characteristics of the oils produced.

**Figure 2:**
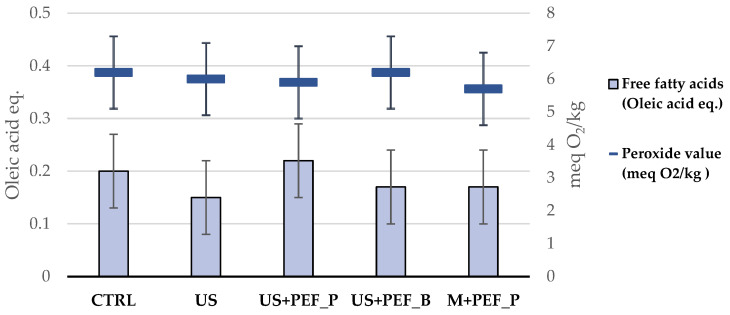
FFA and PV, according to different EVOO production setups (expressed, respectively, as % oleic acid ±U and meq O_2_/kg ± U, where U is the expanded measurement uncertainty with a coverage factor k = 2 and a confidence level of 95%).

Specifically, the analysis of FFA and PV offers critical insight into the chemical stability and quality of EVOO. FFAs are important indicators of the olive’s initial quality and the care taken during the olive-processing phase. A low percentage of FFAs is desirable as it indicates minimal hydrolytic rancidity, which can affect flavour and shelf-life. In the various trials, the percentage of FFAs remained below the maximum threshold recommended by international standards without any statistically significant differences (values between 0.15 and 0.20% oleic acid, ≤0.80%, Table 2 and Figure 2), indicating high-quality olives and extraction processes that minimized enzymatic degradation and hydrolysis.

PVs, on the other hand, measure the primary oxidation products formed in the oil, and serve as a benchmark for the oil’s oxidative freshness as they are precursors to the more complex oxidation products that can spoil the oil. The observed levels for all the employed technologies stayed below the maximum threshold recommended by international standards, without any statistically significant differences (values between 5.7 and 6.2 meq O_2_/kg, ≤20.0 meq O_2_/kg, Table 2 and Figure 2).

UV spectrophotometric investigation provides information on the quality of a fat, its state of preservation and changes brought about by technological processes, indicating the presence of conjugated diene (at 232 nm) and triene systems (at 268 nm) that may result from oxidation processes and/or refining practices. For K232, oils obtained with US and PEF technologies gave slightly higher values than conventional malaxation, but always below the maximum threshold recommended by international standards for EVOOs (1.82 ÷ 1.97 vs. 1.34 ± 0.03, ≤2.5). For K268 and ΔK, no statistically significant differences were observed.

Sensory analyses are only applicable to VOOs, and are crucial for the classification of such oils according to the intensity of perceived defects and fruitiness. Performed by a panel of selected, trained and monitored tasters, these analyses involve olfactory and buccal evaluations to determine the perception of taste (bitter) and tactile sensations (pungent, astringent) which vary in intensity depending on the area of the tongue, palate and throat. The volatile aromatic compounds are perceived in a retronasal olfactory evaluation, while the pungency sensation is also detected by swallowing the oil.

It is well known that most of the taste-active biophenols influence either bitterness or the trigeminal sensation of pungency. During production and storage, biophenols can undergo several changes (qualitative and quantitative) due to oxidation and degradation. As a result, the total phenolic content can be negatively affected, leading to changes in the typical bitter and pungent flavours of fresh EVOO [65]. Furthermore, the “fruitiness” attribute of EVOO is characterised by a fragrant and delicate flavour, typically associated with fresh, healthy olive fruit picked at the peak of ripeness. This sensory quality is further enhanced by notes reminiscent of freshly cut grass, leaves and flowers, as well as green fruits, such as apple, banana and almond, and vegetables such as tomato and artichoke. Consumers usually associate this sensation exclusively with EVOO.

**Figure 3:**
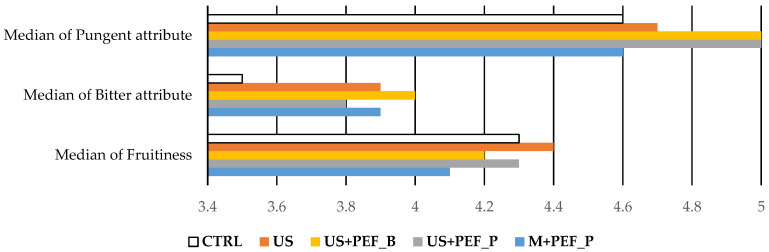
Organoleptic evaluation of the different oils obtained using the conventional process (CTRL) and the application of US and PEF technologies alone or combined (US, US + PEF_P, US + PEF_B, M + PEF_P).

Organoleptic assessment showed a median of fruitiness of between 4.1 (M + PEF_P) and 4.4 (US) with the CTRL sample at 4.3 (Table 2, Figure 3). The median of the bitter attribute was significantly higher for oils extracted using non-conventional techniques, ranging from 3.8 to 4.0, than in the CTRL (3.5). The median of the pungent attribute was higher for oils extracted with US, particularly when both positive and bipolar PEF were applied (5.0 for US + PEF_P and US + PEF_B, 4.7 for US), while the median of the negative attribute with the highest intensity was zero. The almond, green and floral notes of all the oil samples were also perceived. These results determined the classification of the oils obtained as EVOOs (median of fruitiness > 0, median of the negative attribute with the highest intensity = 0, Table 2). It is possible to state that US led to the maximum fruitiness sensations (4.4), accompanied by considerable tastes of bitterness and pungency (3.9 and 4.7). Those latter values, on the other hand, appear to be maximised by the US + PEF protocols (3.8 and 5.0 for positive and 4.0 and 5.0 for bipolar PEF, respectively).

### 3.3. Purity Evaluation of Oil Samples

Purity criteria are important to determine the authenticity of oils and identify any sophistication or fraudulent chemico-physical procedures applied to them. The composition of FAs and sterols is specific to the botanical origin and is related to varietal, environmental and ripening factors. *trans*-FAs can be formed during refining processes or the fraudulent desterolation of the oils, while triterpenic dialcohols, such as uvaol and erythrodiol, should be present in low amounts in olive oil because they are present in the drupe pericarp and, accordingly, in pomace oil. In our previous work (Supplementary Information) [53] it was shown that US had no significant effect on FA composition, FA *trans*-isomers, sterol composition and content, and alcoholic compounds, whereas the ripening stage affected some of them slightly. Moreover, any fraudulent adulteration processes could be excluded, as olives came from the same batch in the same day and the same configuration of the equipment was used to produce all the oil samples, modulating US and PEF units on and off.

For these reasons, these analyses were performed only on the CRTL oil. As shown in Table S1 (Supplementary Information), the composition of FAs and sterols fall within the EVOO specifications [13,29]. Oleic, palmitic, linoleic and stearic acids are the most abundant in the green Coratina CTRL oil (75.1 ± 0.71, 12.66 ± 0.71, 7.17 ± 0.35 and 2.49 ± 0.14 *w*/*w* % + U, respectively, where U is the expanded measurement uncertainty with a coverage factor k = 2 and a confidence level of 95%). On the other hand, β-sitosterol, Δ-5-avenasterol, campesterol, chlerosterol and stigmasterol are the most abundant sterols (85.4 ± 0.7, 6.1 ± 0.2, 3.2 ± 0.3, 1.1 ± 0.1 and 1.0 ± 0.1 *w*/*w* % + U, respectively) with an apparent β-sitosterol value of 94.3 ± 0.9% and a total sterol content of 1146 ± 123 mg/kg.

FA *trans*-isomers (octadecenoic, octadecadienoic and octadecatrienoic) were found below the limits of regulation (≤0.05%), as were erythrodiol and uvaol (1.2 ± 0.4 *w*/*w* % ± U on sterols, ≤4.5%).

### 3.4. Micronutrient Yield

According to the results obtained in our previous work on half-ripening Coratina olives, the main biophenols present in their oil are oleuropein and ligstroside derivatives, oleocanthal, lignans (pinoresinol and acetoxypinoresinol) and flavonoids (luteolin and apigenin derivatives). The amount of polyphenols in the Coratina cultivar (from Puglia) is typically higher than in other Italian cultivars such as Taggiasca and Mattea (from Liguria), as was highlighted in the paper (around 500 mg/kg biophenols vs. 150 mg/kg for half-ripening Taggiasca olives or 290 and 139 mg/kg, respectively, for green Taggiasca and Mattea olives). Polyphenol content was almost unaffected by the non-conventional technologies applied in pilot-scale trials and in medium-sized oil mills, except for in the half-ripening Taggiasca, which showed a slight increase in the total amount of these bioactive compounds.

In the investigation of the innovative large-sized production protocols, US and US + PEF_P resulted in the highest phenol concentrations (1103 and 1057 mg/kg, Table 3, for Tukey comparison, not significantly different), followed by US + PEF_B, CTRL and M + PEF_P (989, 978 and 912 mg/kg, respectively, Table 3). Moreover, from the Tukey post hoc multiple comparisons of means, both the US + PEF_P and US + PEF_B values were not significantly different from the CTRL (b in Table 3, Polyphenols), such as those of US + PEF_B and CTRL from M + PEF_P (c in Table 3, Polyphenols). It is worth noticing that all the samples not only reach the EFSA Health Claim threshold (polyphenol content ≥ 250 mg/kg) [9], but they exceed it by 3 to 4.5 times. Thus, we can state that the green Coratina olives were of excellent quality, and the screened technologies were suitable for valorising the starting material.

Edible oils contain minor compounds such as tocopherols, tocotrienols, carotenoids, and phytosterols. Tocopherols and tocotrienols (α, β, γ, δ), collectively referred to as tocols, are 6-chromanol derivatives with a lateral isoprenoid saturated or unsaturated chain, respectively. They are among the most crucial lipid-soluble antioxidants in both food and the human body, and are present in EVOO in a 90–500 mg/kg range. Due to their antioxidant properties, tocols play a significant role in safeguarding mono- and polyunsaturated FAs (PUFAs) from oxidation, which may also account for the high concentration of these phenolic antioxidants in highly unsaturated edible oils [66].

According to our previous results, the main tocols in half-ripening Coratina olives were α- and γ-tocopherol and α- and γ-tocotrienol. The amount of tocols found in the Coratina cultivar was higher than in the Taggiasca and Mattea varieties (around 300 mg/kg vs. 140 mg/kg for half-ripening Taggiasca olives and 80 and 140 mg/kg, respectively, for green Taggiasca and Mattea olives). Tocol content was almost unaffected by the non-conventional technologies applied in the pilot-scale trials, while they were increased in the medium-sized oil mills when US and PEF were applied.

In the new large-sized industrial trials on green Coratina, M + PEF_P gave the highest tocol concentration (204 mg/kg, Table 3) together with US + PEF_P (199 mg/kg), although they were not significantly different in the Tukey post hoc multiple comparison test (group a in Table 3). They were followed closely by US, US + PEF_B and CTRL values (195, 191 and 189 mg/kg, respectively, Table 2). The Tukey post hoc multiple comparisons of means demonstrated that all the values of tocols from oils obtained under non-conventional technologies were significantly different from that of the CTRL (c in Table 3). It is worth noting that total tocol content is higher in half-ripening Coratina olives than in green ones, as occurred for Taggiasca at different ripening stages.

Remarkably, US and its combination with positive PEF resulted in promising polyphenol and tocol increases (+12 and +8%, respectively, for polyphenols, +3 and +5%, respectively, for tocols).

### 3.5. Preliminary Evaluation of Micronutrient Stability in EVOOs

In EVOO, phenolic compounds predominantly exist as aglycones and various secoiridoid derivatives, such as oleuropein and ligstroside. These compounds are known for their antioxidant properties and play a crucial role in extending the shelf-life of virgin olive oils [67]. Over time, these phenolics are subject to both qualitative and quantitative changes as they decompose and react via oxidative processes during storage [68]. Tocopherols and tocotrienols can be affected by the system in which they are present, as well as by the conditions under which they are stored and processed [69], and by the cooking procedures employed [70].

In the different studies carried out both on commercial oils stored under market conditions and samples incubated under accelerated conditions (40 °C), α-tocopherol was partially lost, although it was above the established limit of 18 mg/kg for the 4th health claim throughout the entire testing period [10,71]. On the other hand, it was suggested that only samples with an initial content of tyrosol derivatives over 1000 mg/kg could maintain the health claim almost indefinitely over the EVOO shelf life.

**Figure 4:**
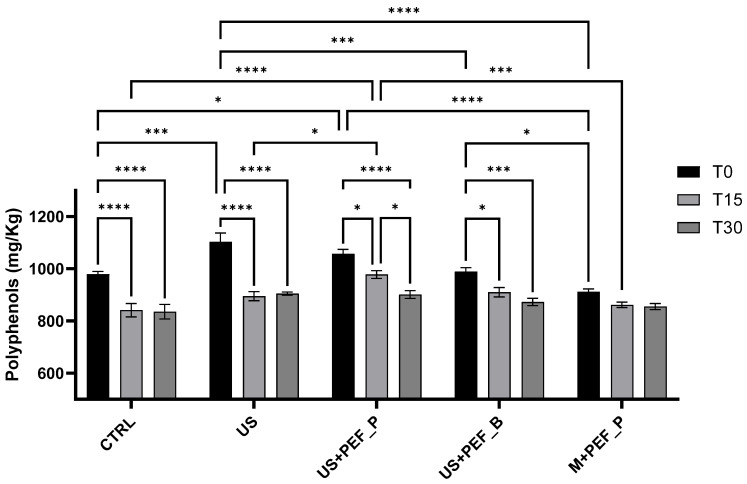
Significant differences in polyphenol content between EVOOs obtained using different techniques (CTRL, US, US + PEF_P, US + PEF_B, M + PEF_P) immediately after production (T0) and samples kept in the climatic chamber (ASLT, light, 40 °C) for T15 and T30 (two-way ANOVA with Graph-Pad Prism 10.2.3) Statistical significance is indicated by *p*-values as follows: *p* < 0.05 (*), *p* < 0.001 (***), and *p* < 0.0001 (****).

**Figure 5:**
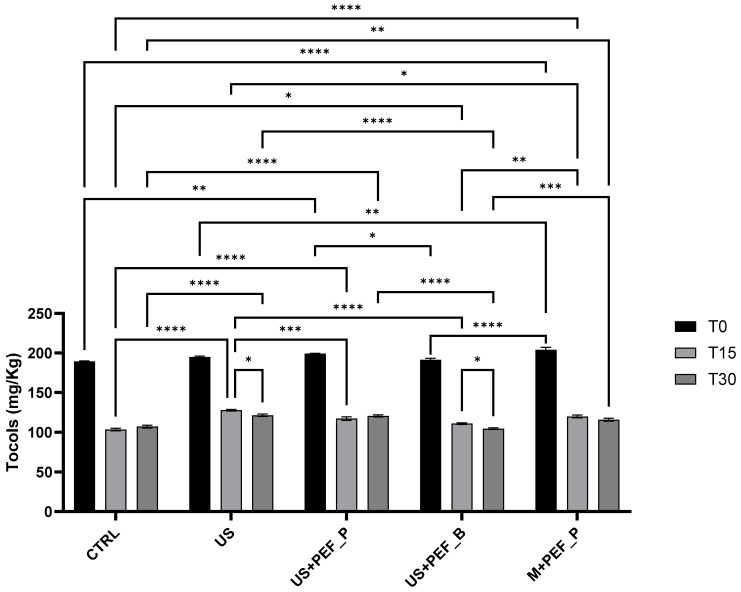
Significant differences in tocol content between EVOO obtained using different techniques (CTRL, US, US + PEF_P, US + PEF_B, M + PEF_P) immediately after production (T0) and samples kept in the climatic chamber (ASLT, light, at 40 °C) for T15 and T30 (two-way ANOVA with Graph-Pad Prism). Highly significant differences (****) between T0 and T15 and T0 and T30 were removed from graphic for reasons of clarity. Statistical significance is indicated by *p*-values as follows: *p* < 0.05 (*), *p* < 0.01 (**), *p* < 0.001 (***)and *p* < 0.0001 (****).

To understand the effect of extraction techniques and time, and their possible interactions, a two-way ANOVA was performed on total polyphenols in EVOO immediately after production, and the samples were kept in the climatic chamber for 15 and 30 days. Two factors were considered, technique with five levels (CTRL, US, US + PEF_P, US + PEF_B, M + PEF_P) and time with 3 levels (T0, T15, and T30). Both technique and time were highly significant (*p* < 0.001, responsible for 26.6% and 52.6% of the total variance, respectively), while their interaction was found to be very significant (*p* = 0.0023, responsible for 10.8% of the total variance). Tukey’s multiple comparisons test was applied to highlight the significant differences (α = 0.05), which have been summarized in Figure 4 and Figure 5. Means, confidence intervals and Tukey’s groups for time into technique and technique into time are presented in Table 4.

At T0, as described in Section 3.3, US showed the highest polyphenol content, which was significantly different from those of CTRL, US + PEF_B (0.001 < *p* < 0.0001) and M + PEF_P (*p* < 0.0001), while similar to that of US + PEF_P (*p* > 0.05). Moreover, the US + PEF_P treatment was significantly different from the CTRL (0.05 < *p* < 0.01) and M + PEF_P (*p* < 0.0001), while similar to the US + PEF_B (*p* > 0.05) (Figure 4; groups a–d in Table 4, column T0).

For the CTRL and US protocols, the amount of total polyphenols at T15 was highly significantly different (*p* < 0.0001), while for treatments combined with PEF there was a slight (US + PEF_P or B, 0.05 < *p* < 0.01) or no difference (M + PEF_P, *p* > 0.05) (Figure 4; groups 1 and 2 in Table 4, within rows). At T30, all treatments but M + PEF_P (*p* > 0.05) showed significant differences from T0 (groups 1–3 in Table 4, within rows). When comparing samples at T15 and T30, no differences were found except for a slightly lower value for US + PEF_P (0.05 < *p* < 0.01) (Figure 4; group 3 in Table 4, within row). At T15, the only significant differences were between US + PEF_P and CTRL (*p* < 0.0001), M + PEF_P (0.001 < *p* < 0.0001) and US (0.05 < *p* < 0.01) (Figure 4; groups a and b in Table 4, column T15). At T30, there were no significant differences between samples (group a in Table 4, column T30).

From this screening, it can be deduced that US helped to enhance the extraction of phenolic components from olive pomace. The technique provided the highest amount of total polyphenols in EVOO (near 1100 mg/kg), alone and when combined with positive PEF, from the same Coratina olive batch, compared to CTRL, PEF alone and bipolar PEF (910 ÷ 990 mg/kg). Although polyphenol values become comparable for all treatments (835 ÷ 905 mg/kg) after a few months of shelf life, PEF seemed to play an important role in stabilizing them, enhancing overall EVOO quality throughout the first period of ageing. Furthermore, it is worth noticing that the EFSA “health” requirements for identification as EVOO were abundantly fulfilled.

The two-way ANOVA performed on total tocols in EVOO immediately after production and at T15 and T30 demonstrated that both extraction technique and time were highly significant (*p* < 0.001, responsible for 2.5% and 96.5% of the total variance, respectively), together with their interaction (*p* < 0.001, responsible for 0.7% of the total variance). Tukey’s multiple comparisons test was applied to highlight the significant differences (α = 0.05), which have been summarised in Figure 5. Means, confidence intervals and Tukey’s groups for time into technique and technique into time are presented in Table 5.

At T0, as described in Section 3.3, M + PEF_P and US + PEF_P showed the highest tocol content. US + PEF_P was significantly different from CTRL (0.005 < *p* < 0.001) and US + PEF_B (0.05 < *p* < 0.01), while similar to M + PEF_P and US (*p* > 0.05) (Figure 5; groups a–c in Table 5, column T0).

For all techniques, highly significant differences between T0 and T15, and between T0 and T30 were found (*p* < 0.0001), and were eliminated from Figure 5 for reasons of clarity (group 1–3 in Table 5, within rows). Comparing samples at T15 and T30, no differences were found except for US and US + PEF_B (0.05 < *p* < 0.01) (Figure 5; group 3 in Table 4, within rows).

At T15, the CTRL sample showed the lowest value of total tocols, which was highly significantly different from those of US, US + PEF_P and M + PEF_P (*p* < 0.0001) and only slightly from that of US + PEF_B (0.05 < *p* < 0.01) (Figure 5; groups a–d in Table 6, column T15). The highest tocol content was found for the US sample followed by US + PEF_P (0.001 < *p* < 0.0001) and M + PEF_P (0.05 < *p* < 0.01) (Figure 5; groups a and b Table 6, column T15). At T30, the EVOO obtained using US, US + PEF_P and M + PEF_P were found to be comparable (group a in Table 5, column T30). The CTRL sample showed significantly lower values of total tocols from US and US + PEF_P (*p* < 0.0001) and M + PEF_P (0.005 < *p* < 0.001), while these values were comparable to those of US + PEF_B (*p* > 0.05) (Figure 5; group b in Table 5, column T30).

In general, the different trials generate differences in terms of tocol concentrations, with the minimum amount being observed in the CTRL sample (189 mg/kg) and the best performance achieved by the PEF system (near 200 mg/kg). The stability trend suggests that US and positive PEF, alone or in combination, help to reduce tocol degradation, giving higher values at T30.

### 3.6. Preliminary Assessment of Stability of Organoleptic Characteristics of Obtained EVOO

The organoleptic properties related to the flavour of EVOO are due to the presence of a volatile fraction and some minor compounds, such as phenolics. These properties can be greatly affected by several factors, such as the olive variety, growing conditions and ripening stage, giving each EVOO a particular flavour. Furthermore, they can be affected by oxidation processes and temperature throughout the oil’s shelf-life, and are vulnerable to degradation. These alterations in the composition of EVOO generate changes in its organoleptic properties [72].

**Figure 6:**
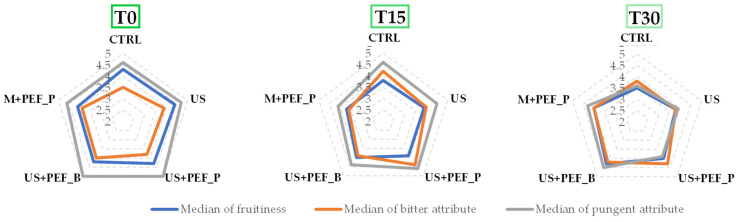
Organoleptic evaluation, stability trends at T0, T15 and T30.

The integration of US and PEF technologies has also shown promising effects on the organoleptic qualities of EVOO when considering shelf-life evolution, with median levels of fruitiness, bitterness and pungency being sustained across a range of sampling intervals (Figure 6).

For example, the PEF setup demonstrated a notable consistency in fruitiness at T30, suggesting a robustness against the sensory degradation that is typically observed over time. This may be linked to the enhanced cell disruption that is caused by PEF, which facilitates the improved release and retention of the volatile compounds that are responsible for these sensory attributes. Moreover, while all of the oils underwent natural declines in sensory attributes, due to oxidation, the combination setups (US + PEF_P and US + PEF_B) consistently led to higher scores in bitterness and pungency, usually due to phenolics, which are important for consumer acceptance and the perceived quality of EVOO. These findings suggest that the simultaneous application of US and PEF might be particularly effective in preserving the robust flavours that are highly prized in premium olive oils. Numerical data are collected in Tables S2–S6.

In conclusion, the importance of integrating innovative technologies, such as US and PEF, in EVOO production presents a promising approach to enhancing both the quality and stability of the oil. The consistent performance of US + PEF_P across the board further suggests that PEF technology, particularly when integrated with acoustic cavitation, not only improves the initial extraction efficiency and quality, but can effectively mitigate the natural degradation of sensory attributes, contributing to the oil’s resistance to oxidative changes over time, offering significant benefits for commercial-scale operations.

### 3.7. Technical and Economic Feasibility

In addition to nutritional benefits and production efficiency, it is crucial to assess the technical and economic feasibility of implementing US and PEF technologies in EVOO production. This assessment considers the capital investment, operational costs, scalability and potential return on investment associated with these innovative processes. By examining these factors, we aim to provide a comprehensive understanding of the viability and benefits of employing US and PEF technologies in the olive oil industry.

As shown in Table 7, the current plant can process at the following rates:

- 24 tons/day of olive paste with the conventional process with 4 malaxation tanks;
- 36 tons/day of olive paste with the ultrasonic process;
- 36 tons/day of olive paste with the US process combined with the PEF (both positive, “P”, and bipolar, “B”).

In assessing the industrial scale-up of alternative technologies for olive-oil processing, in particular US and PEF, the data clearly show several advantages and challenges. The implementation of US and US + PEF as a replacement for the traditional malaxing step significantly increases the daily processing capacity of olive paste from 24 tons/day, using conventional methods, to 36 tons/day, with a constant +50% [73]. This increase potentially reduces the bottleneck effects traditionally associated with the malaxation dwell time and represents a significant increase in productivity. Specifically, the replacement technologies streamline the flow through the decanters, which historically has been a limiting factor in scaling up production (Table 6). Essentially, US can process 50% more olive paste than the conventional process as the limiting factor is the maximum flow rate of the separation stage.

**Table 6:** Outlook for EVOO process setups.

Process	Malax. (Time)	Process Flow Rate * (ton/h)	Daily Processed Olive Paste (ton/Day)	Oil Yields (*w*/*w* %)	Oil Prod. (ton/Day) **	Energy Cons. (kW/Day)	Energy Cons. (kW/ton Oil)
CTRL	30 min	1.0	24	14.6	3.5	1944	555
US	-	1.5	36	14.0	5.0	1905	381
US + PEF_P	-	1.5	36	14.2	5.1	1934	379
US + PEF_B	-	1.5	36	13.8	4.9	1934	395
M + PEF_P	30 min	1.0	24	14.4	3.5	1980	566

* Decanter flow rate: 1.5 ton/h. Process flow rate calculated taking into account the malaxation time (when required). ** Calculated on 24 h/day.

**Figure 7:**
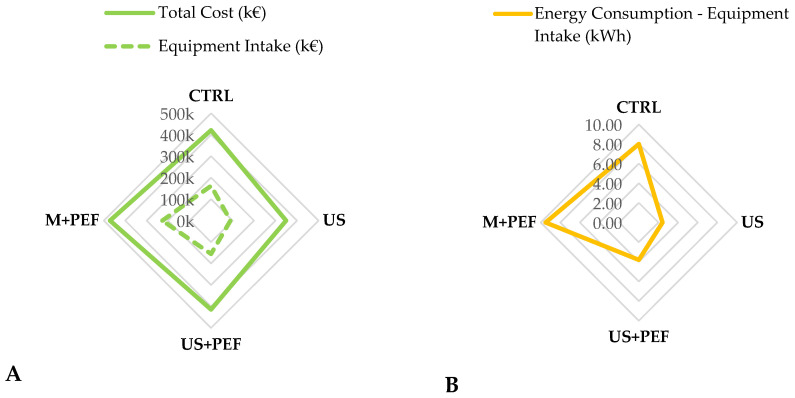
(**A**): Set-up cost comparison; (**B**): energy intake according to the key equipment. Production stream has been normalized to 36 ton/day.

In general, the adoption of advanced technologies, such as US and PEF, either alone or in combination, shows distinct economic and energy-consumption patterns that significantly influence the feasibility of industrial scale-up in olive oil extraction (see Figure 7 and Figures S2–S5). Firstly, the transition from conventional methods to those incorporating US and PEF technologies not only modifies the equipment landscape, but also affects the cost and energy dynamics. The base-cost analysis reveals that the most economical scenario is when US is utilized alone, with costs totalling EUR 349,000 (savings of EUR 72,000, with respect to the conventional approach) with an energy consumption of 79.4 kWh (−5.6 kWh). By contrast, the combination of US and PEF (polar mode) escalates the total cost to EUR 414,000 (modest saving of EUR 7000, with respect to the conventional approach), although the energy reduction is roughly maintained, with energy consumption at 80.8 kWh (−4.2 kWh) (Figure 7).

This indicates that, while PEF integration incurs higher up-front costs, it does not proportionately increase energy consumption, suggesting efficient integration with US in terms of energy use. Moreover, a comparison with the PEF-only setup, which integrates traditional malaxation and costs EUR 471,000 with an energy consumption of 86.5 kWh, underscores the cost-effectiveness and energy efficiency of US-based methods. The PEF-only setup, with a higher cost, offers an energy consumption rate that is comparable to CTRL, positioning it as the least favourable option solely from an economic standpoint (Figure 7). Numerical data are collected in Tables S7–S10.

In general, these technological implementations not only avoid the need for malaxers, but also reduce the overall energy consumption per kg of produced oil, which is reported to be 34.6% lower in US and 34.8% lower in US + PEF_P processes compared to the conventional method (Table 6 and Table 7). Economically speaking, the US method alone presents the best case for scale-up due to its significantly lower equipment and operational costs compared to all other tested configurations. The substantial cost and energy savings make it an attractive option for EVOO producers aiming for process intensification with controlled overheads.

**Table 7:** EVOO production technology comparison, reported as % increase/decrease (+/−), compared to CTRL process.

Process	Total Polyphenols (%)	Total Tocols (%)	Oil Prod. (%)	Energy Cons. per kg Oil (%)	Total Equipment Cost (%)
US	+12.6	+3.2	+42.9	−34.6	−17.1
US + PEF_P	+7.9	+5.3	+45.7	−34.8	−1.7
US + PEF_B	-	-	+40.0	−32.1	−1.7
M + PEF_P	−6.9	+7.9	-	+1.8	+11.9

From a quality perspective, the integration of US and PEF technologies also influences the biochemical composition of the olive oil produced. The US process, both with and without PEF, results in higher levels of total phenolics and tocols; a key factor in determining the antioxidant properties and shelf-life of the oils. The increases in total phenolics and tocols of +12.6% and +3.2%, respectively, for US, and +7.9% and +5.3%, respectively, for US + PEF_P, are a significant improvement in oil quality (Table 7).

However, despite these positive outcomes, the adaptation of these technologies on an industrial scale must be approached with caution. The non-differential cost of upgrading to a more powerful decanter, which is essential for both US and conventional processes, underlines the importance of strategic investment in technology, in line with production goals and financial constraints.

## 4. Conclusions

This study successfully implemented novel technologies in olive oil mills to develop an efficient large-sized industrial process for the continuous extraction of healthier EVOOs that are enriched with bioactive compounds, such as polyphenols and tocols. By integrating US and pulsed electric field (PEF) technologies into a single continuous-flow setup, we eliminated the need for traditional malaxation. This innovative setup, which combines acoustic cavitation and electroporation, not only improved daily oil production by nearly 45%, but also enriched the EVOO nutritional profile with significant increases in polyphenols (8–12%) and tocols (3–5%), while also providing better stability in these parameters in the first months after production. These enhancements elevate the quality, market value and shelf-life of EVOO. At 30 days AAT, all the oil samples recorded maintained the initial classification category of EVOO with good organoleptic properties, showing values of median fruitiness and bitter and pungent attributes always above 3.5, without negative attributes, and total amounts of tocols and polyphenols that meet the EFSA 4th and 5th “health” claims. Additionally, the elimination of kneading during malaxation resulted in significant energy savings (approximately 35%). Thus, the introduction of continuous-flow US and PEF technologies represents a significant advancement in the EVOO industry, offering substantial benefits to both producers and consumers by meeting the growing demand for healthier, nutrient-enriched products.

Finally, the presented data support the technological advancement and economic viability of using US and US + PEF_P in EVOO production. This approach not only enhances productivity and oil quality, but also optimizes energy use, representing a promising shift towards more sustainable and efficient agricultural practices. However, the choice of technology must align with specific operational goals, while balancing the trade-offs between initial investments and on-going energy costs. Our detailed analysis of these configurations provides a comprehensive basis for decision-making in the deployment of these innovative technologies in industrial settings. Future studies should focus on long-term operational data and the impact of these technologies on consumer acceptance and market dynamics.

## Figures and Tables

**Table 1:** PEF conditions.

Parameters	US + PEF_P (3)	US + PEF_B (4)	M_PEF_P (5)
Voltage; Pulse; Frequency	[10 kV; 30 µs; 12 Hz]	[10 kV; 30 µs; 5 Hz]	[10 kV; 30 µs; 17 Hz]
Electric Field	2 kV/cm	2.0 kV/cm	2 kV/cm
Total Energy	5.1 kJ/kg	5.1 kJ/kg	5.1 kJ/kg
Power	1.5 kW	1.5 kW	1.5 kW

**Table 2:** Analyses of EVOOs produced using classical oil mill (CTRL) and non-conventional techniques (US and positive or bipolar PEF) from the green Coratina variety.

Analysis	Method	Parameter (Meas. Unit)	CTRL	US	US + PEF_P	US + PEF_B	M + PEF_P	EVOO Spec. ^#^
FFA (expressed as oleic acid)	[56]	% ± U **^§^**	0.20 ± 0.07	0.15 ± 0.07	0.22 ± 0.07	0.17 ± 0.07	0.17 ± 0.07	≤0.80
PV	[57]	meq O_2_/kg ± U **^§^**	6.2 ± 1.1	6.0 ± 1.1	5.9 ± 1.1	6.2 ± 1.1	5.7 ± 1.1	≤20.0
Insoluble impurities	[58]	*w*/*w* % ± U **^§^**	<0.01 ± 0.01	0.01 ± 0.01	<0.01 ± 0.01	<0.01 ± 0.01	<0.01 ± 0.01	≤0.10
Moisture and volatile matter (B)	[59]	*w*/*w* % ± U **^§^**	0.15 ± 0.04	0.15 ± 0.05	0.16 ± 0.05	0.14 ± 0.05	0.14 ± 0.05	≤0.20
UV spectrophotometric analysis	[60]	K232 (±SD) *	1.34 ± 0.03	1.97 ± 0.03	1.87 ± 0.01	1.82 ± 0.09	1.83 ± 0.07	≤2.50
		K268 (±SD) *	0.18 ± 0.03	0.19 ± 0.02	0.20 ± 0.01	0.19 ± 0.005	0.17 ± 0.01	≤0.22
		ΔK (±SD) *	−0.002 ± 0.000	−0.001 ± 0.003	0.000 ± 0.001	−0.001 ± 0.003	−0.001 ± 0.000	≤0.01
Organoleptic assessment	[55]	Category	EVOO	EVOO	EVOO	EVOO	EVOO	
		Median of fruitiness (Mf)	4.3	4.4	4.3	4.2	4.1	>0.0
		Median of bitter attribute	3.5	3.9	3.8	4.0	3.9	-
		Median of pungent attribute	4.6	4.7	5.0	5.0	4.6	-
		Median of the negative attribute with the highest intensity (Md)	0.0	0.0	0.0	0.0	0.0	=0
		Notes perceived with the highest intensity:	Almond, green, floral	Almond, green, floral	Almond, green, floral	Almond, green, floral	Almond, green, floral	-

(^#^) REG. CE 2568/91 Annex 1 and IOC Trade standards [13,29]. (^§^) U = Expanded measurement uncertainty with a coverage factor k = 2 and a confidence level of 95%. * SD = standard deviation.

**Table 3:** Total polyphenol and tocol (tocopherols and tocotrienols) content in oil samples, expressed as mg per kg of oil.

Analysis	Method	Compound (Meas. Unit)	CTRL	US	US + PEF_P	US + PEF_B	M + PEF_P
Tocopherols and tocotrienols	[61]	α-tocopherol (the only detectable) (mg/kg) (CL 0.95, SE 1.74) *	189 ^c^ (185 ÷ 193)	195 ^bc^ (191 ÷ 199)	199 ^ab^ (195 ÷ 203)	191 ^bc^ (188 ÷ 195)	204 ^a^ (200 ÷ 208)
Polyphenols	[60]	mg/kg (CL 0.95, SE 19.6) * (RRF 4.95)	980 ^bc^ (936 ÷ 1023)	1103 ^a^ (1059 ÷ 1146)	1057 ^ab^ (1014 ÷ 1101)	989 ^bc^ (946 ÷ 1033)	912 ^c^ (868 ÷ 955)

* CL = Confidence level; SE = Standard Error. ^a,b,c^ Tukey post hoc comparisons. *p* value adjustment: Tukey method for comparing a family of five estimates, significance level used: α = 0.05. CL: 0.95.

**Table 4:** Total polyphenol content in oil samples at T0, T15 and T30 in the climatic chamber, expressed as mg/kg of oil.

Technique	T0 (mg/kg) *	T15 (mg/kg) *	T30 (mg/kg) *	Loss % T15	Loss % T30
CTRL	980 ^cd, 1^ (943 ÷ 1017)	841 ^b, 2^ (805 ÷ 878)	836 ^a, 2^ (799 ÷ 873)	−14.1	−14.7
US	1103 ^a, 1^ (1066 ÷ 1140)	895 ^b, 2^ (858 ÷ 932)	905 ^a, 2^ (868 ÷ 942)	−18.8	−17.9
US + PEF_P	1057 ^ab, 1^ (1021 ÷ 1094)	978 ^a, 2^ (941 ÷ 1015)	901 ^a, 3^ (865 ÷ 938)	−7.5	−14.7
US + PEF_B	989 ^bc, 1^ (953 ÷ 1026)	910 ^ab, 2^ (874 ÷ 947)	873 ^a, 2^ (836 ÷ 910)	−8.0	−11.8
M + PEF_P	912 ^d, 1^ (875 ÷ 948)	862 ^b, 1^ (825 ÷ 899)	856 ^a, 1^ (819 ÷ 892)	−5.4	−6.1

* Confidence level used = 0.95, Standard Error = 18, obtained via two-way ANOVA (R software). ^a,b,c,d^ Tukey post hoc comparisons within columns. ^1,2,3^ Tukey post hoc comparisons within rows. Conf-level adjustment: Bonferroni method for five estimates, *p* value adjustment: Bonferroni method for 10 tests, for technique into time, while three estimates and three tests for time into technique. Significance level used: α = 0.05.

**Table 5:** Total tocol content in oil samples at T0, T15 and T30 in the climatic chamber, expressed as mg/kg of oil.

Technique	T0 (mg/kg) *	T15 (mg/kg) *	T30 (mg/kg) *	Loss % T15	Loss % T30
CTRL	189 ^c, 1^ (185÷194)	103 ^d, 2^ (99÷108)	107 ^b, 2^ (103÷112)	−45.5	−43.4
US	195 ^b,c 1^ (190÷199)	128 ^a, 2^ (123÷132)	121 ^a, 3^ (117÷126)	−34.4	−37.9
US + PEF_P	199 ^a,b, 1^ (195÷204)	117 ^b,c, 2^ (113÷122)	121 ^a, 2^ (116÷125)	−41.2	−39.2
US + PEF_B	191 ^c, 1^ (187÷196)	111 ^c, 2^ (106÷115)	105 ^b, 3^ (100÷109)	−41.9	−45.0
M + PEF_P	204 ^a, 1^ (199÷209)	120 ^b, 2^ (125÷124)	116 ^a, 2^ (111÷120)	−41.2	−43.1

* Confidence level used = 0.95, Standard Error = 1.65, obtained using two-way ANOVA (R software). ^a,b,c,d^ Tukey post hoc comparisons within columns. ^1,2,3^ Tukey post hoc comparisons within rows. Conf-level adjustment: Bonferroni method for five estimates, *p* value adjustment: Bonferroni method for 10 tests, for technique into time, while three estimates and three tests for time into technique. Significance level used: α = 0.05.

## Data Availability

The original contributions presented in the study are included in the article/Supplementary Materials, further inquiries can be directed to the corresponding author.

## Supplementary Materials

The following supporting information can be downloaded at: https://www.mdpi.com/article/10.3390/foods13162613/s1, Figure S1: (a) EVOO plant, overview. US + PEF system configuration, which was used with PEF device off for trial “US”, with both US and PEF (positive mode) on for trial “US + PEF_P”, with both US and PEF (bipolar mode) on for trial “US + PEF_B”, used with US devices off for trial M + PEF_P (malaxation first and then PEF in positive mode). (b) US industrial reactor. (c) A: PEF generator and treatment chamber; B: PEF generator connection to decanting unit and controller screen, Figure S2: Pareto charts relative to CTRL trial. A: total equipment cost; B: Energy consumption, Figure S3: Pareto charts relative to US trial. A: total equipment cost; B: Energy consumption, Figure S4: Pareto charts relative to US + PEF trial. A: total equipment cost; B: Energy consumption, Figure S5: Pareto charts relative to M + PEF trial. A: total equipment cost; B: Energy consumption, Table S1: Fatty acids and sterols composition of EVOO produced by classical oil mill (CONTROL), Table S2: Analysis of olive oils produced by classical oil mill (CONTROL) from green Coratina variety (External certified laboratories, Unito laboratories), Table S3: Analysis of olive oils produced by the application of ultrasound (US) from green Coratina variety (External laboratories, Unito laboratories), Table S4: Analysis of olive oils produced by the application of combined ULTRASOUND and positive PEF (US + PEF_P) from green Coratina variety (External laboratories, Unito laboratories), Table S5: Analysis of olive oils produced by the application of combined ULTRASOUND and bipolar PEF (US + PEF_B) from green Coratina variety (external laboratories, Unito laboratories), Table S6: Analysis of olive oils produced by the application of positive PEF (M + PEF_P) from green Coratina variety (External laboratories, Unito laboratories), Table S7: Costs of major equipment and Energy consumption–Conventional Oil extraction [CTRL], Table S8: Costs of major equipment and Energy consumption–Ultrasound oil extraction [US Trial], Table S9: Costs of major equipment and Energy consumption–Ultrasound oil extraction combined with Pulsed Electric field (polar mode) [US + PEF_P Trial], Table S10: Costs of major equipment and Energy consumption–Pulsed Electric field (polar mode) extraction combined with malaxation [M + PEF_P Trial].
