# Supplementary material for: Industrial Production of Bioactive Nutrient-Enhanced Extra Virgin Olive Oil under Continuous-Flow Ultrasound and Pulsed Electric Field Treatment
Source: Foods. 2024 Aug 20;13(16):2613. doi: 10.3390/foods13162613 (PMC11353453; doi:10.3390/foods13162613)
Supplement: Supplementary file 1 [file foods-13-02613-s001.zip › foods-3127304-supplementary.pdf]

Supporting Information

Industrial Production of Bioactive Nutrient-Enhanced Extra Virgin Olive Oil under Continuous-Flow Ultrasound and Pulsed Electric Field Treatment

Luisa Boffa [1], Emanuela Calcio Gaudino [1], Giorgio Grillo [1], Arianna Binello [1], Giorgio Capaldi [1], Duarte Rego [2], Marcos Pereira [2] and Giancarlo Cravotto [1,*]

[1] Department of Drug Science and Technology, University of Turin, Via P. Giuria 9, 10125 Turin, Italy; luisa.boffa@unito.it (L.B.); emanuela.calcio@unito.it (E.C.G.); giorgio.grillo@unito.it (G.G.); arianna.binello@unito.it (A.B.); giorgio.capaldi@unito.it (G.C.)

[2] EnergyPulse Systems, Est Paco Lumiar Polo Tecnológico Lt3, 1600-546 Lisbon, Portugal; duarte.rego@energypulsesystems.com (D.R.); marcos.pereira@energypulsesystems.com (M.P.)

* Correspondence: giancarlo.cravotto@unito.it; Tel.: +39-011-670-7183

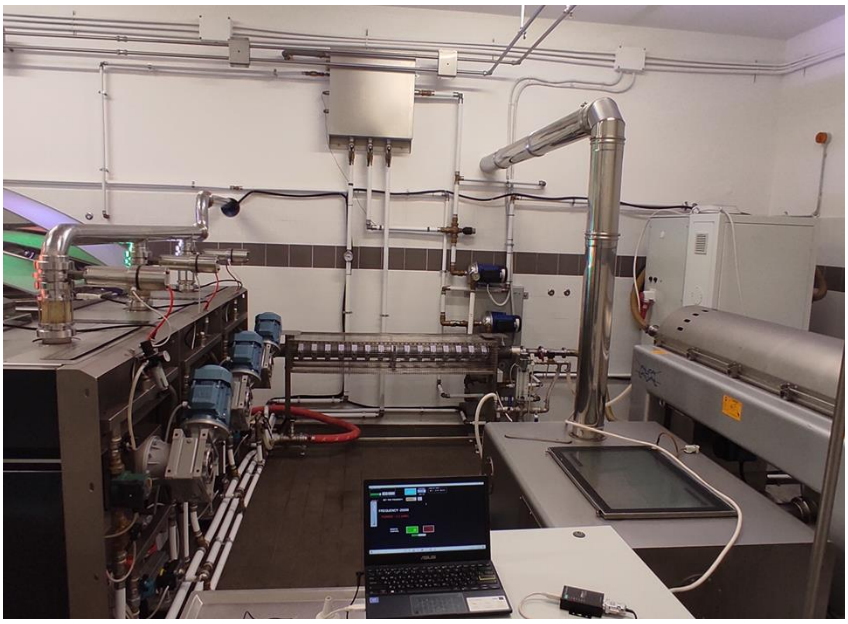

(a)

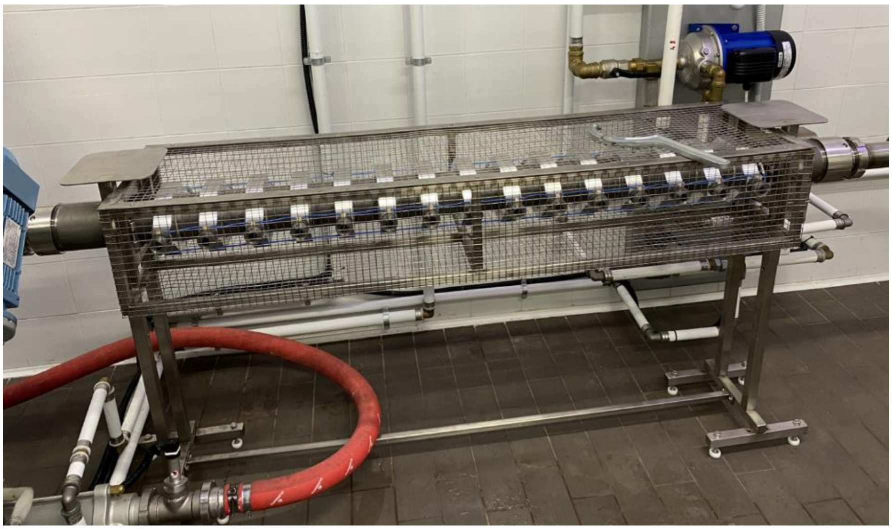

(b)

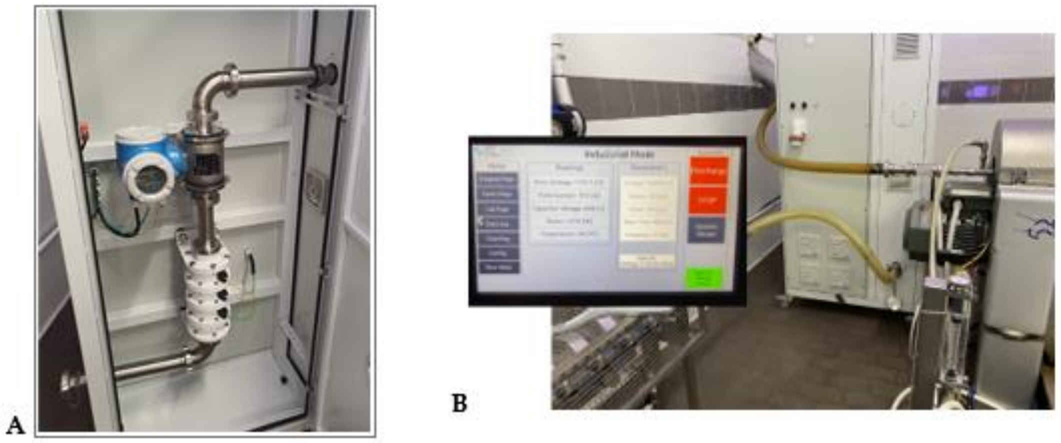

(c)

Figure S1: (a)EVOO plant, overview. US+PEF system configuration, which was used with PEF device off for trial "US", with both US and PEF (positive mode) on for trial "US+PEF_P", with both US and PEF (bipolar mode) on for trial "US+PEF_B", used with US devices off for trial M+PEF_P (malaxation first and then PEF in positive mode). **(b)** US industrial reactor. **(c)** A: PEF generator and treatment chamber; B: PEF generator connection to decanting unit and controller screen.

Table S1. Fatty acids and sterols composition of EVOO produced by classical oil mill (CONTROL).

**Analysis**	**Method**	**Compound or test (Mean percentage ± U)** §	**Green Coratina CRTL**	**EVOO Spec.**#
Fatty acids composition	COl/T.20/Doc. No 33/Rev. 1 2017	Myristic acid	0.01±0.01	< 0.03
		Pentadecanoic acid	<0.01	-
		Palmitic acid	12.66±0.71	7.50-20.00
		Palmitoleic acid	0.76±0.07	0.30-3.50
		Heptadecanoic acid	0.04±0.02	≤ 0.40
		Heptadecenoic acid	0.06±0.02	≤ 0.60
		Stearic acid	2.49±0.14	0.50-5.00
		Oleic acid	75.11±0.71	55.00-83.00
		Linoleic acid	7.17±0.35	2.50-21.00
		Arachidic acid	0.41±0.07	≤ 0.60
		Eicosenoic acid	0.34±0.07	≤ 0.50
		Linolenic acid	0.71±0.07	≤ 1.00
		Behenic acid	0.11±0.07	≤ 0.20
		Lignoceric acid	0.05±0.03	≤ 0.20
Fatty acids *trans*-isomers	COl/T.20/Doc. No 33/Rev. 1 2017	Octadecenoic acids	0.05±0.01	≤ 0.05
		Octadecadienoic + octadecatrienoic acids	0.01±0.01	≤ 0.05
Sterol composition and content and alcoholic compounds	COI/T.20/ Doc. No 26/Rev. 5 2020	Cholesterol	0.1±0.1	≤ 0.5
		tR Brassicasterol	< 0.1±0.1	≤ 0.1
		24-Metilencolesterol	0.1±0.1	-
		Campesterol	3.2±0.3	≤ 4.0

Campestanol	0.1±0.1	-
Stigmasterol	1.0±0.1	< campesterol
Δ-7-campesterol	< 0.1±0.1	-
Δ-5,23-stigmastadienol	< 0.1±0.1	-
Chlerosterol	1.1±0.1	-
β-sitosterol	85.4±0.7	-
Sitostanol	0.9±0.2	-
Δ-5-avenasterol	6.1±0.2	-
Δ-5,24-stigmastadienol	0.8±0.1	-
Δ-7-stigmastenol	0.4±0.1	≤ 0.5
Δ-7-avenasterol	0.7±0.1	
Apparent β-sitosterol	94.3±0.9	≥ 93.0
Total sterols (mg/kg)	1146±123	≥ 1000
Erythrodiol + uvaol (% ±U) §	1.2±0.4	≤ 4.5

(#) REG. CE 2568/91 Annex 1 and Doc. COI/T.15/NC No 3/Rev. 14-2019.

(§) U = Expanded measurement uncertainty with a coverage factor k = 2 and a confidence level of 95%.

Table S2. Analysis of olive oils produced by classical oil mill (CONTROL) from green **Coratina variety** (External certified laboratories, Unito laboratories).

**Analysis**	**Method**	**Compound or test (Meas. Unit)**	**Green Coratina**			**EVOO Spec.**[#]
			**Control T0** [a]	**Control T15**	**Control T30**	
**Organoleptic assessment (T0 and 15, T30)**	COI/T.20/Doc. No 15/Rev. 10 2018	Category	EVOO	EVOO	EVOO	**-**
		Median of fruitiness (Mf)	**4.3**	**3.8**	**3.5**	> 0.0
		Median of bitter attribute	3.5	4.2	3.8	-
		Median of pungent attribute	4.6	4.6	3.6	-
		Median of the negative attribute with the highest intensity (Md)	**0.0**	**0.0**	**0.0**	= 0
		Notes perceived with the highest intensity	Almond, green, floral	Almond, green	-	-
**Tocopherols and tocotrienols**	IUPAC 1992	α-tocopherol (the only detectable) (mg/kg) (±SD)	**189.3±1.6**	**103.5±3.0**	**105.7±3.1**	-
**Polyphenols**	COI/T.20/ Doc. No 29/Rev.1/2017	mg/kg (±SD) (RRF 4.95)	**980±17**	**841±44**	**836±48**	**-**

[a] The T0 analysis herein reported are referred to the oil directly obtained after extraction. This oil was subsequently subjected to a filtration step before being stored in the climatic chamber (for the times listed in the table of 15 and 30 days) without being further analysed. (#) Limits in Annex I of COMM. DEL. REG. (EU) 2022/2104+COMM. IMPL. REG. (EU) 2022/2105.

Table S3. Analysis of olive oils produced by the application of ultrasound (US) from green **Coratina variety** (External laboratories, Unito laboratories)

**Analysis**	**Method**	**Compound or test (Meas. Unit)**	**Green Coratina**			**EVOO Spec.**[#]
			**US T0** [a]	**US T15**	**US T30**	
**Organoleptic assessment (T0 and 15, T30)**	COI/T.20/Doc. No 15/Rev. 10 2018	Category	EVOO	EVOO	EVOO	**-**
		Median of fruitiness (Mf)	**4.4**	3.9	3.8	> 0.0
		Median of bitter attribute	3.9	4.0	3.8	-
		Median of pungent attribute	**4.7**	4.5	3.9	-
		Median of the negative attribute with the highest intensity (Md)	**0.0**	0.0	0.0	= 0
		Notes perceived with the highest intensity:	Almond, green, floral	Almond, green	-	-
**Tocopherols and tocotrienols**	IUPAC 1992	α-tocopherol (the only detectable) (mg/kg) (±SD)	**194.9±2.2**	**127.8±1.7**	**119.9±2.7**	-
**Polyphenols**	COI/T.20/ Doc. No 29/Rev.1/2017	mg/kg (±SD) (RRF 4.95)	**1103±59**	**895±30**	**905±10**	**-**

[a] The T0 analysis herein reported are referred to the oil directly obtained after extraction. This oil was subsequently subjected to a filtration step before being stored in the climatic chamber (for the times listed in the table of 15 and 30 days) without being further analysed. (#) Limits in Annex I of COMM. DEL. REG. (EU) 2022/2104+COMM. IMPL. REG. (EU) 2022/2105.

Table S4. Analysis of olive oils produced by the application of combined ULTRASOUND and positive PEF (US+PEF_P) from green **Coratina variety** (**External laboratories**, **Unito** laboratories)

**Analysis**	**Method**	**Compound or test (Meas. Unit)**	**Green Coratina**			**EVOO Spec.**[#]
			**US+PEF_P T0** [a]	**US+PEF_P T15**	**US+PEF_P T30**	
**Organoleptic assessment (T0 and 15, T30)**	COl/T.20/Doc. No 15/Rev. 10 2018	Category	EVOO	EVOO	EVOO	**-**
		Median of fruitiness (Mf)	**4.3**	**3.9**	**4.0**	> 0.0
		Median of bitter attribute	**3.8**	4.4	4.3	-
		Median of pungent attribute	**5.0**	4.6	3.9	-
		Median of the negative attribute with the highest intensity (Md)	**0.0**	**0.0**	**0.0**	= 0
		Notes perceived with the highest intensity:	Almond, green, floral	Almond, green	-	-
**Tocopherols and tocotrienols**	IUPAC 1992	α-tocopherol (the only detectable) (mg/kg) (±SD)	**199.2±0.7**	**117.2±4.0**	**119.2±2.5**	-
**Polyphenols**	COl/T.20/ Doc. No 29/Rev.1/2017	mg/kg (±SD) (RRF 4.95)	**1057±30**	**978±26**	**901±26**	**-**

[a] The T0 analysis herein reported are referred to the oil directly obtained after extraction. This oil was subsequently subjected to a filtration step before being stored in the climatic chamber (for the times listed in the table of 15 and 30 days) without being further analysed. (#) Limits in Annex I of COMM. DEL. REG. (EU) 2022/2104+COMM. IMPL. REG. (EU) 2022/2105.

Table S5. Analysis of olive oils produced by the application of combined ULTRASOUND and bipolar PEF (US+PEF_B) from green **Coratina variety** (**external laboratories**, **Unito** laboratories)

**Analysis**	**Method**	**Compound or test (Meas. Unit)**	**Green Coratina**			**EVOO Spec.**[#]
			**US+PEF_B T0** [a]	**US+PEF_B T15**	**US+PEF_B T30**	
**Organoleptic assessment (T0 and 15, T30)**	COI/T.20/Doc. No 15/Rev. 10 2018	Category	EVOO	EVOO	EVOO	**-**
		Median of fruitiness (Mf)	**4.2**	**4.0**	**4.3**	> 0.0
		Median of bitter attribute	4.0	3.9	4.2	-
		Median of pungent attribute	5.0	4.4	4.5	-
		Median of the negative attribute with the highest intensity (Md)	**0.0**	**0.0**	**0.0**	= 0
		Notes perceived with the highest intensity:	Almond, green, floral	Almond, green	-	-
**Tocopherols and tocotrienols**	IUPAC 1992	α-tocopherol (the only detectable) (mg/kg) (±SD)	**191.4±3.0**	**110.9±1.7**	**103.2±1.7**	-
**Polyphenols**	COI/T.20/ Doc. No 29/Rev.1/2017	mg/kg (±SD) (RRF 4.95)	**989±26**	**910±31**	**873±24**	**-**

[a] The T0 analysis herein reported are referred to the oil directly obtained after extraction. This oil was subsequently subjected to a filtration step before being stored in the climatic chamber (for the times listed in the table of 15 and 30 days) without being further analysed. (#) Limits in Annex I of COMM. DEL. REG. (EU) 2022/2104+COMM. IMPL. REG. (EU) 2022/2105.

Table S6. Analysis of olive oils produced by the application of positive PEF (M+PEF_P) from green **Coratina variety** (External laboratories, Unito laboratories)

Analysis	Method	Compound or test (Meas. Unit)	Green Coratina			EVOO Spec.[#]
			M+PEF_P T0 [a]	M+PEF_P T15	M+PEF_P T30	
**Organoleptic assessment (T0 and 15, T30)**	COI/T.20/Doc. No 15/Rev. 10 2018	Category	EVOO	EVOO	EVOO	**-**
		Median of fruitiness (Mf)	**4.1**	**3.7**	**4.0**	> 0.0
		Median of bitter attribute	3.9	3.6	4.0	-
		Median of pungent attribute	4.6	4.1	4.3	-
		Median of the negative attribute with the highest intensity (Md)	**0.0**	**0.0**	**0.0**	= 0
		Notes perceived with the highest intensity:	Almond, green, floral	Almond, green	-	-
**Tocopherols and tocotrienols**	IUPAC 1992	α-tocopherol (the only detectable) (mg/kg) (±SD)	**204.1±5.4**	**119.7±3.1**	**114.2±3.2**	-
**Polyphenols**	COI/T.20/ Doc. No 29/Rev.1/2017	mg/kg (±SD) (RRF 4.95)	**912±20**	**862±18**	**856±20**	**-**

[a] The T0 analysis herein reported are referred to the oil directly obtained after extraction. This oil was subsequently subjected to a filtration step before being stored in the climatic chamber (for the times listed in the table of 15 and 30 days) without being further analysed. (#) Limits in Annex I of COMM. DEL. REG. (EU) 2022/2104+COMM. IMPL. REG. (EU) 2022/2105.

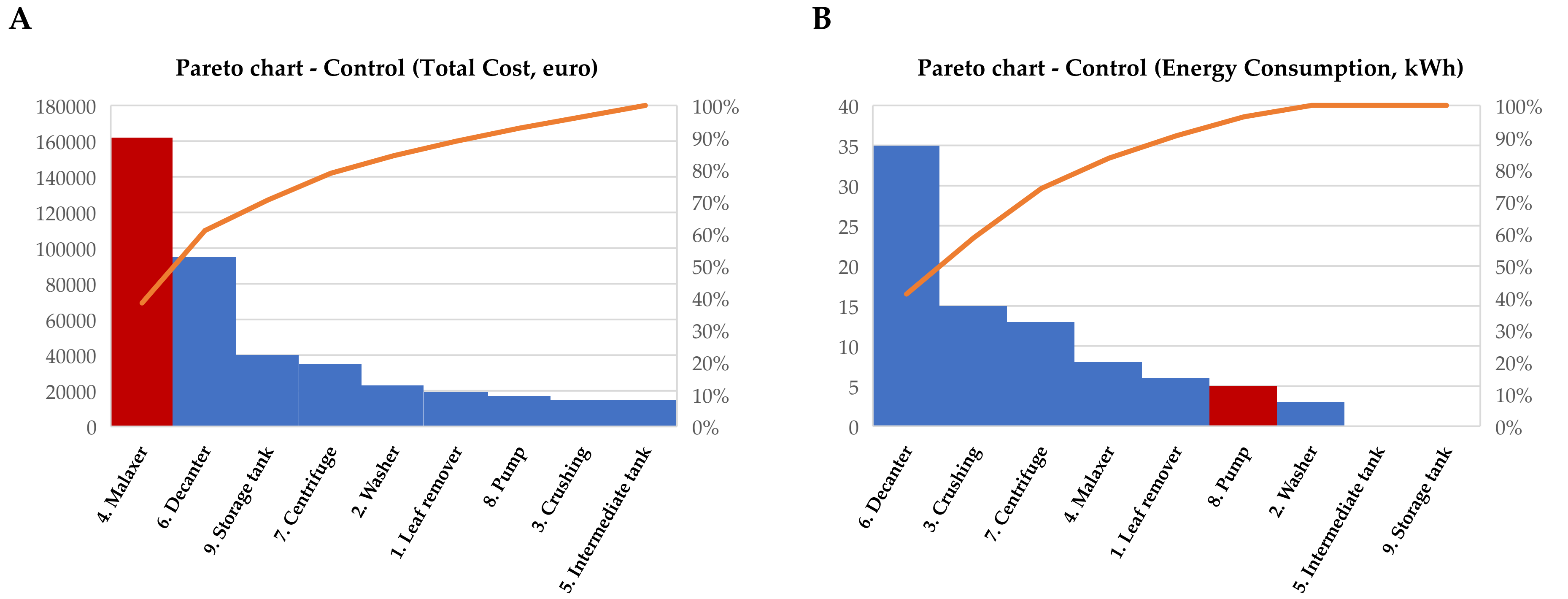

Figure S2: Pareto charts relative to CTRL trial. A: total equipment cost; B: Energy consumption.

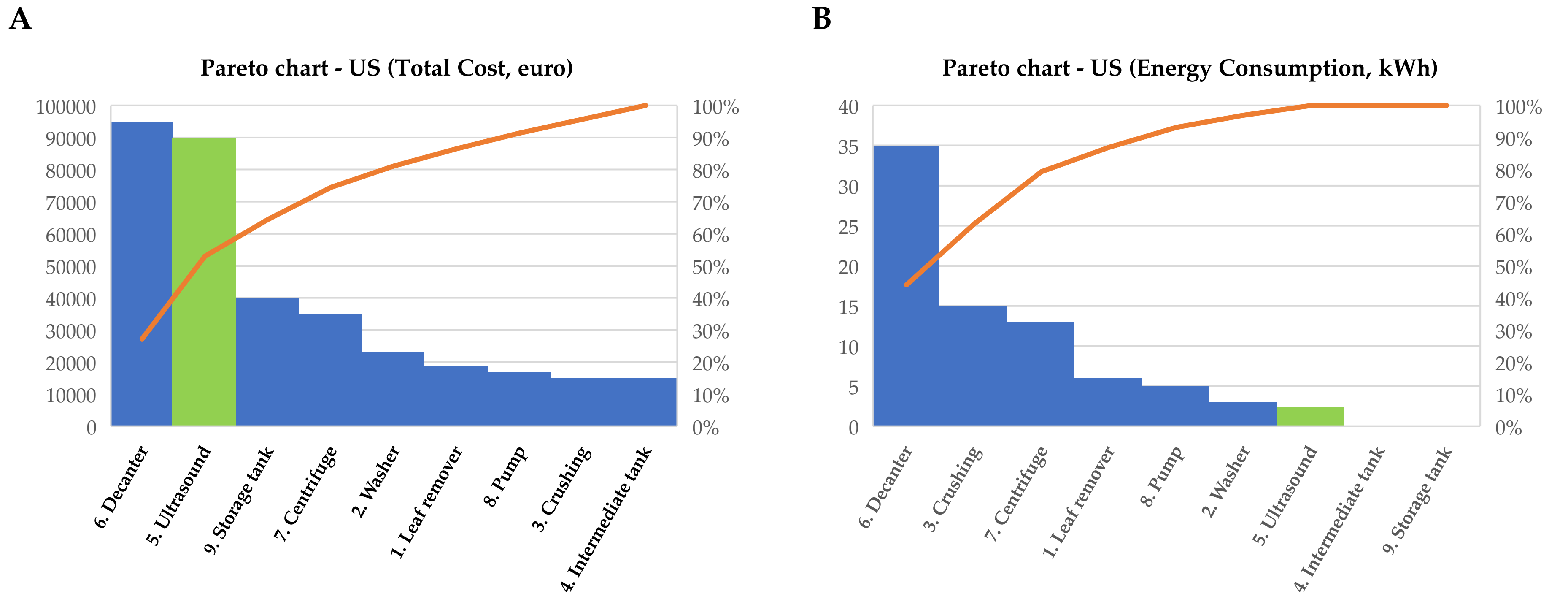

Figure S3: Pareto charts relative to US trial. A: total equipment cost; B: Energy consumption.

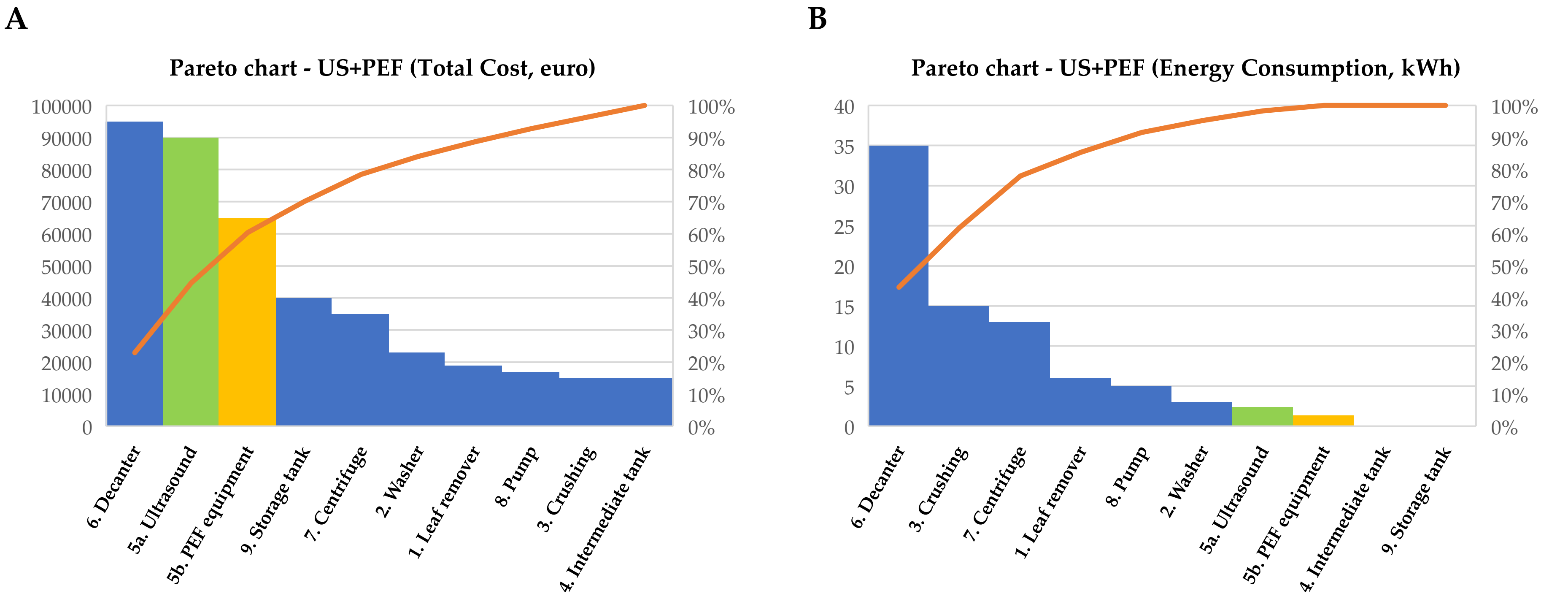

Figure S4: Pareto charts relative to US+PEF trial. A: total equipment cost; B: Energy consumption.

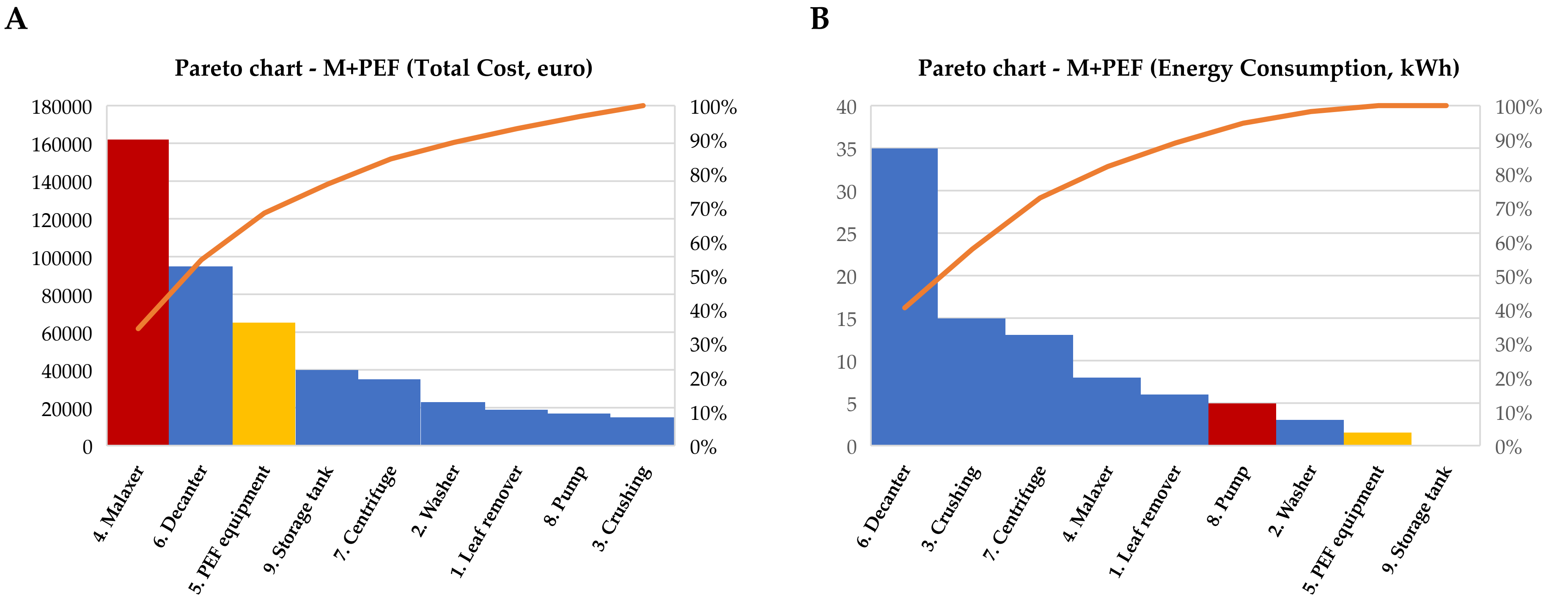

Figure S5: Pareto charts relative to M+PEF trial. A: total equipment cost; B: Energy consumption.

Table S7. Costs of major equipment and Energy consumption – Conventional Oil extraction [CTRL]

Item	Base unit cost (Euro)	Number of units	Total base cost (Euro)	Energy Consumption (kWh)
1. Leaf remover	19000	1	19000	6
2. Washer	23000	1	23000	3
3. Crushing	15000	1	15000	15
4. Malaxation container	27000	6	162000	8
5. Intermediate tank	15000	1	15000	0
6. Decanter	95000	1	95000	35
7. Centrifuge	35000	1	35000	13
8. Pump	17000	1	17000	5
9. Storage tank	40000	1	40000	0
**Total**			421000	85

Table S8. Costs of major equipment and Energy consumption – Ultrasound oil extraction [US Trial]

Item	Base unit cost (Euro)	Number of units	Total base cost (Euro)	Energy Consumption (kWh)
1. Leaf remover	19000	1	19000	6
2. Washer	23000	1	23000	3
3. Crushing	15000	1	15000	15
4. Intermediate tank	15000	1	15000	0
5- Ultrasound	90000	1	90000	2.4
6. Decanter	95000	1	95000	35
7. Centrifuge	35000	1	35000	13
8. Pump	17000	1	17000	5
9. Storage tank	40000	1	40000	0
**Total**			349000	79.4

Table S9. Costs of major equipment and Energy consumption – Ultrasound oil extraction combined with Pulsed Electric field (polar mode) [US+PEF_P Trial]

Item	Base unit cost (Euro)	Number of units	Total base cost (Euro)	Energy Consumption (kWh)
1. Leaf remover	19000	1	19000	6
2. Washer	23000	1	23000	3
3. Crushing	15000	1	15000	15
4. Intermediate tank	15000	1	15000	0
5-Ultrasound	90000	1	90000	2.4
5-PEF equipment	65000	1	65000	1.4
6. Decanter	95000	1	95000	35
7. Centrifuge	35000	1	35000	13
8. Pump	17000	1	17000	5
9. Storage tank	40000	1	40000	0
**Total**			414000	80.8

Table S10. Costs of major equipment and Energy consumption – Pulsed Electric field (polar mode) extraction combined with malaxation [M+PEF_P Trial]

Item	Base unit cost (Euro)	Number of units	Total base cost (Euro)	Energy Consumption (kWh)
1. Leaf remover	19000	1	19000	6
2. Washer	23000	1	23000	3
3. Crushing	15000	1	15000	15
4. Malaxation container	27000	6	162000	8
5-PEF equipment	65000	1	65000	1.5
6. Decanter	95000	1	95000	35
7. Centrifuge	35000	1	35000	13
8. Pump	17000	1	17000	5
9. Storage tank	40000	1	40000	0
**Total**			471000	86.5
